# Identification of Potent and Selective Inhibitors of *Acanthamoeba*: Structural Insights into Sterol 14α-Demethylase as a Key Drug Target

**DOI:** 10.1021/acs.jmedchem.4c00303

**Published:** 2024-04-29

**Authors:** Tatiana Y. Hargrove, David C. Lamb, Zdzislaw Wawrzak, Marcus Hull, Steven L. Kelly, F. Peter Guengerich, Galina I. Lepesheva

**Affiliations:** † Department of Biochemistry, Vanderbilt University School of Medicine, Nashville, Tennessee 37232, United States; ‡ Faculty of Medicine, Health and Life Science, Swansea University, Swansea SA2 8PP, U.K.; § Synchrotron Research Center, Life Science Collaborative Access Team, Northwestern University, Argonne, Illinois 60439, United States; ∥ Vanderbilt Institute of Chemical Biology, Nashville, Tennessee 37232, United States; ⊥ Center for Structural Biology, Vanderbilt University, Nashville, Tennessee 37232, United States

## Abstract

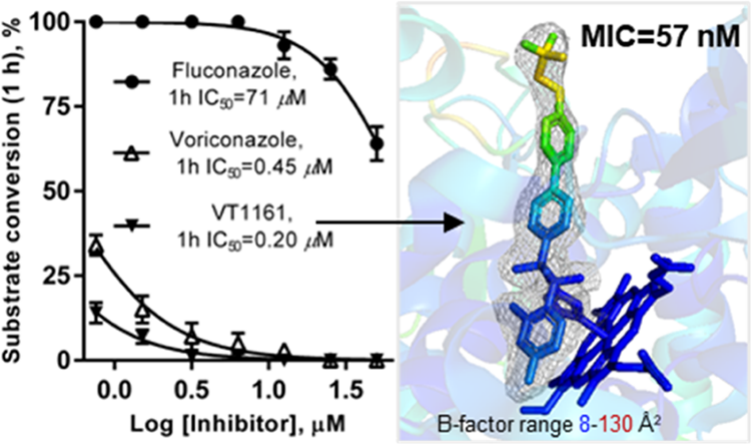

*Acanthamoeba* are free-living pathogenic protozoa that cause blinding keratitis, disseminated infection, and granulomatous amebic encephalitis, which is generally fatal. The development of efficient and safe drugs is a critical unmet need. *Acanthamoeba* sterol 14α-demethylase (CYP51) is an essential enzyme of the sterol biosynthetic pathway. Repurposing antifungal azoles for amoebic infections has been reported, but their inhibitory effects on *Acanthamoeba* CYP51 enzymatic activity have not been studied. Here, we report catalytic properties, inhibition, and structural characterization of CYP51 from *Acanthamoeba castellanii*. The enzyme displays a 100-fold substrate preference for obtusifoliol over lanosterol, supporting the plant-like cycloartenol-based pathway in the pathogen. The strongest inhibition was observed with voriconazole (1 h IC_50_ 0.45 μM), VT1598 (0.25 μM), and VT1161 (0.20 μM). The crystal structures of *A. castellanii* CYP51 with bound VT1161 (2.24 Å) and without an inhibitor (1.95 Å), presented here, can be used in the development of azole-based scaffolds to achieve optimal amoebicidal effectiveness.

## Introduction

*Acanthamoeba* is a genus of free-living single-celled eukaryotic organisms (Amoebozoa) found ubiquitously in the environment (soil, airborne dust, fresh and salt waters) and in human-made items such as contact lenses, medical devices, air conditioners, and cooling towers.^1,2^ Several of them (especially *Acanthamoeba castellanii* and *Acanthamoeba polyphaga*) are opportunistic pathogens for humans and animals. In healthy humans, they most commonly cause a sight-threatening infection of the cornea, known as blinding keratitis, mostly associated with the use of contact lenses.^3^ In immunocompromised patients, however, *Acanthamoeba* can cause granulomatous amebic encephalitis (GAE), a rare and generally fatal infection of the brain and spinal cord, as well as the disseminated infection that affects the skin, sinuses, lungs, and other organs independently or in combination.^4,5^ Moreover, *Acanthamoeba* have been shown to harbor a variety of microbial human pathogens that subsequently cause diseases: viruses, including adenovirus, poliovirus, poxviruses, and enterovirus; fungi, e.g., *Cryptococcus neoformans*, *Histoplama capsulaus*, and *Blastomyces dermatitidis*; and bacteria, e.g., *Mycobacterium tuberculosis*, *Legionella pneumophila*, *Pseudomonas aeruginosa*, and *Staphylococcus aureus*.^1,6−10^ The incidence of infections is growing because of the large number of contact lens wearers and the increasing number of immunocompromised patients.

Diagnosis of *Acanthamoeba* eye infections is challenging, and the few available treatments are lengthy and not fully effective. Current treatment regimens usually include topical disinfectant agents (e.g., chlorhexidine, biguanides, diamidines) and miltefosine, which are highly toxic, and, occasionally, azole antifungals.^11−14^ If bacteria are also associated with the infection, the addition of antibiotics is recommended (e.g., neomycin or chloramphenicol).^15^ There is no recommended treatment for GAE, and the majority of cases are diagnosed at the post-mortem stage. Even if diagnosed early, the lack of available antiamoebic drugs (particularly with the ability to cross the blood–brain barrier) results in poor prognosis, with the mortality rate being 97–98%.^16,17^ There have been, however, a few reported cases of successful treatment, and they involved the use of different antimicrobial drug combinations, including amphotericin B and several antifungal azoles.^15^

In antifungal therapy, the modes of action of amphotericin B and azole drugs relate to their selective effects on fungal sterols. Amphotericin B depletes ergosterol from fungal cell membranes, and the azoles block ergosterol biosynthesis by inhibiting the essential cytochrome P450 (P450) enzyme sterol 14α-demethylase (CYP51, or erg11) of the fungal sterol pathway. *Acanthamoeba* are known to synthesize ergosterol as their primary sterol, and unlike some other protozoa, they are unable to take up and utilize exogenous sterols from their environment.^18^ The *Acanthamoeba* ergosterol biosynthetic pathway, however, has been found to be different from that in fungi. While in yeast and fungi squalene-2,3-epoxide is cyclized into lanosterol, *A. polyphaga* was shown to cyclize it into cycloartenol, analogous to the pathway found in plants and algae.^18,19^ This biochemical peculiarity can be explained by the early evolutionary divergence of amoebae from the main line of eukaryotic descent. Based on rRNA sequences, it has been estimated that *Acanthamoeba* diverged sometime between the divergence of yeast (∼1.2 × 10^9^ years ago) and the divergence of plants and animals (∼1 × 10^9^ years ago).^15^ Raederstorff and Rohmer reported that in *A. polyphaga*, cycloartenol is converted into 24-methylenecycloartanol and then into obtusifoliol, with no lanosterol or lanosterol intermediates detectable, even by sensitive radiochemical methods.^18^ These findings were later challenged by Thompson et al., who claimed to detect not only lanosterol but also 4,4-dimethyl-cholesta-8,14,24-trienol (the product of lanosterol 14α-demethylation) in *A. castellanii*.^20^ At approximately the same time, the *A. castellanii* sterols were also examined in the Nes laboratory, and the compounds were found to be similar to those previously identified in *A. polyphaga*,^18^ thus suggesting the same cycloartenol-based ergosterol pathway in both organisms.^21^

We have analyzed the *A. castellanii* sterol 14α-demethylase (CYP51) in the current project, focusing on the structure/function relationship and inhibition. Strict preferences of the enzyme toward obtusifoliol as the substrate support the ergosterol pathway proposed by Raederstorff *et al.* and Kidane *et al*.^18,21^ Among the tested inhibitors, the strongest potency was displayed by a newly approved clinical antifungal drug, tetrazole-based oteseconazole (VT1161). The inhibitory effect on the reconstituted CYP51 activity *in vitro* correlated well with the results of the cellular growth inhibition assay. Consequently, tetrazole chemical scaffolds similar to that of oteseconazole, coupled with *Acanthamoeba* CYP51 structure–function analysis, provide a starting point for the generation of new derivatives for the effective treatment of life-threatening and drug-resistant *Acanthamoeba* infections.

## Results and Discussion

### Sequence Analysis

The primary sequence of *A. castellanii* CYP51 (NCBI accession number XP_004334294) consists of 486 amino acid residues, including the 43-residue long N-terminal membrane anchor (up to Pro44), molecular weight ∼56 kDa. A BLASTP search in the NCBI database revealed the closest ortholog in *A. polyphaga* (only one residue variation, Y441C, helix L). The next closest sequences are from red algae, *Galdieria* spp. (49% identity), the biflagellate cryptomonad *Guillardia theta*, and zooflagellate *Thecamonas trahens* (48% identity), then by green algae (e.g., *Chlorella*, *Prototheca*, 45–43%) and plants (43–41%). This correlates with the notion that amoebae are more closely related to plants than to fungi or animals,^22^ as well as the hypothesis that *Acanthamoeba* evolved and diversified very early in the course of eukaryotic evolution^23^ as the identity with the CYP51 ortholog from the brain-eating amoeba *Naegleria fowleri* is only 38%. The sequence identity is lower to animal CYP51s (sea urchin 34%, human 33%) than, surprisingly, to another group of protists, Trypanosomatidae (*Trypanosoma brucei* 31%). The lowest identities *Acanthamoeba* ortholog has with CYP51s are from fungi (e.g., the yeast *Cryptococcus neoformans*, 30%, and filamentous ascomycetes *Aspergillus fumigatus* B, 29%, *A. fumigatusA*, 27%).

**Figure 1:**
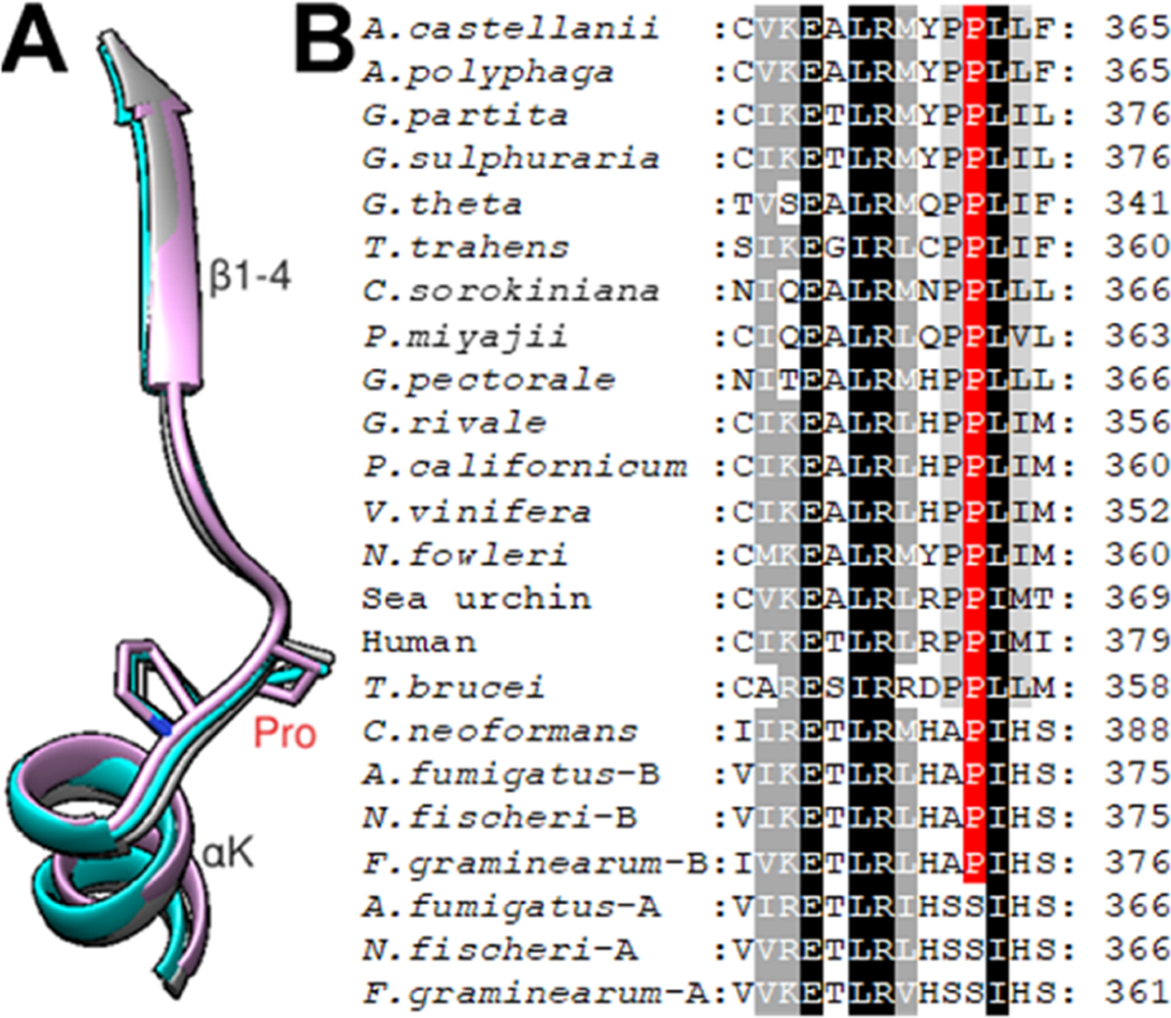
B-type CYP51 signature proline (marked red). (A) In the superimposed *N. fowleri* (cyan, 7RQT), *T. brucei* (plum, 3G1Q), and human (gray, 6UEZ) structures. (B) The proline-surrounding fragment of the aligned CYP51 sequences.

In terms of the A/B-type of CYP51s, the *A. castellanii* ortholog clearly belongs to the B-type (as do all sterol 14α-demethylases from the organisms that have a single *cyp*51 gene^24^). It contains the CYP51B-type signature proline (P362) in the helix K – β1–4 strand connecting area (Figure 1A). Based on our previous CYP51 structural analysis, this proline residue increases the rigidity of this portion of the CYP51 active site cavity,^25^ thus being one of the structural features that make CYP51 enzymes a highly successful target for chemical inhibition.^24^ In contrast, in the (drug-inducible^26^) CYP51A sequences, known to be associated with fungal azole resistance,^26−28^ there is always serine in this position^29^ (Figure 1B, complete sequence alignment of a larger number of CYP51 proteins can be found in Supporting Information, Figure S1). Substitution of this proline with serine in the Y strain *T. cruzi* CYP51A (P355S) was experimentally proven to lower the enzyme drug sensitivity.^25^

### Spectral Characteristics

**Figure 2:**
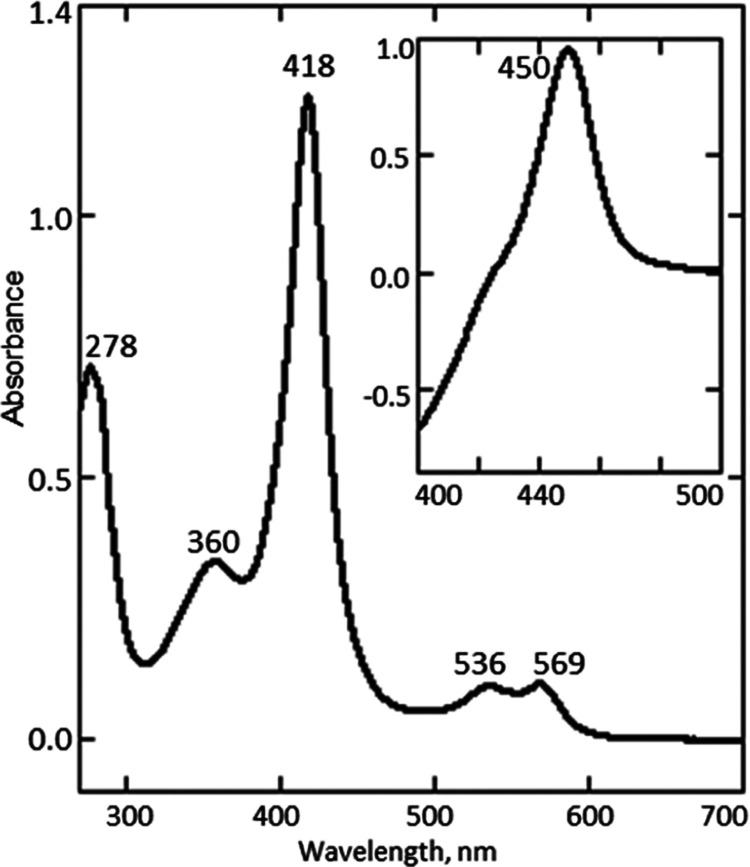
Optical absorbance of purified *A. castellanii* CYP51. The absolute spectrum of ferric protein and the difference spectrum of the ferrous CO-complex (inset). The P450 concentration is 10 μM. The absolute spectrum of the ferric sample was taken first, then the protein was reduced with sodium dithionite and the CO gas was bubbled through the cuvette after taking the baseline.

The UV–visible absolute absorbance spectrum of the purified *A. castellanii* CYP51 (Figure 2) is typical of a ferric, low-spin water-bound P450 with the Soret band maximum at 418 nm, a Δ*A*_393–470_/Δ*A*_418–470_ ratio of 0.35 and a spectrophotometric index (*A*_418_/*A*_278_) of 1.65. The heme iron is readily reduced by sodium dithionite, and the difference spectrum of the ferrous carbon monoxide complex has an absorbance maximum at 450 nm with no (denatured) cytochrome P420 detected.

### Substrate Preferences and Catalytic Activity

**Figure 3:**
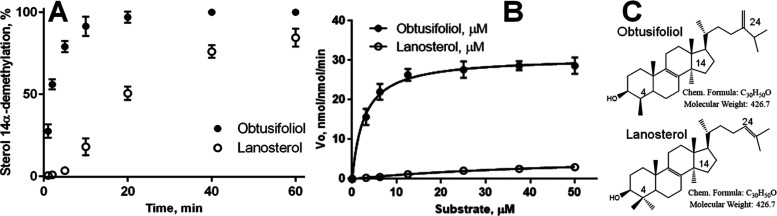
Catalytic activity of *A. castellanii* CYP51 toward obtusifoliol and lanosterol. (A) Time course of substrate conversion (37 °C, 0.5 μM P450, 2 μM CPR, 50 μM sterol). (B) Michaelis–Menten curves (calculated from the 1 min reactions with obtusifoliol and 20 min reactions with lanosterol). Rates (*V*_0_) are shown as nmol product formed per min per nmol P450. The experiments were performed in triplicate; the results are presented as mean ± SD. (C) Structural formulas.

As expected on the basis of the presence of the plant-like phenylalanine in the CYP51 signature 1 sequence in helix-B′,^30^ (Phe116, marked in green in Figure S1), *A. castellanii* CYP51 prefers C4-monomethylated obtusifoliol as the substrate. Moreover, in contrast with the CYP51 ortholog from *N. fowleri*,^31^ the substrate preferences toward obtusifoliol are much stricter, supporting the plant-like postsqualene portion of the pathway.^18^ The differences between the time-course curves of *A. castellanii* CYP51 14α-demethylation of obtusifoliol and lanosterol (Figure 3A) suggest some hindrance to lanosterol binding, with a much lower *k*_cat_ and higher *K*_m_ values calculated from the Michaelis–Menten curves (Figure 3B), with the catalytic efficiency (i.e., *k*_cat_/*K*_m_, the specificity constant) of *A. castellanii* CYP51 for obtusifoliol being 11.4 μM^–1^ min^–1^ (∼100-fold higher than that for lanosterol (0.12 μM^–1^ min^–1^)) (Table 1) and thus the enzyme is much more plant-like than the CYP51 ortholog from *N. fowleri* (3.2 and 1.3 μM^–1^ min^–1^), respectively.^31^

**Table 1:** Steady-State Kinetic Parameters of *A. castellanii* CYP51

substrate	*k*_cat_ (min^–1^)	*K*_m_ (μM)	*k*_cat_/*K*_m_
obtusifoliol	30.8 ± 0.7	2.7 ± 0.3	11.4
lanosterol	6.1 ± 1.2	52 ± 15	0.12

### Spectral Responses to the Binding of the Sterol Substrates and Heme-Coordinating Heterocyclic Ligands

**Figure 4:**
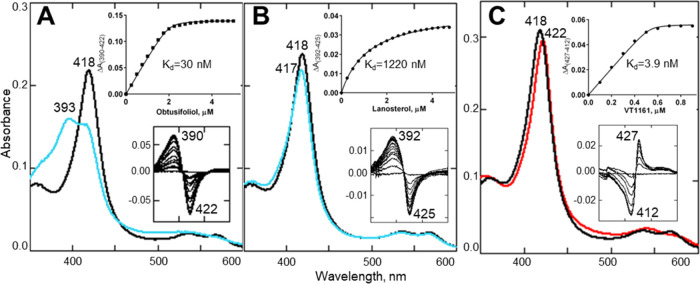
Spectral responses to the binding of sterol substrates (A) obtusifoliol, (B) lanosterol, 2 μM P450, optical path 1 cm, and (C) a tetrazole-based inhibitor VT1161, 0.5 μM P450, optical path length 5 cm. Absolute absorption spectra before (black) and after the titrations (blue and red, respectively). Inset: The titration curves and the corresponding difference absorption spectra used in each analysis. Structural formulas are shown in Figure 6. The titration curves for the binding of other heterocyclic ligands are presented in Figure S2.

Spectral titrations of *A. castellanii* CYP51 with obtusifoliol and lanosterol produced, respectively, ∼60 and 15% low- to high-spin transition in the heme iron (known as a type I P450 spectral response,^32^ or a blue shift in the Soret band maximum due to expulsion of the iron-coordinating water molecule so that the iron becomes pentacoordinated). The calculated apparent dissociation constants, *K*_d_, were 0.03 and 1.22 μM (Figure 4A,B). The >40-fold difference in the *K*_d_ values of the enzyme-sterol complexes is consistent with obtusifoliol being a better substrate and a binding ligand of a higher affinity. This is in contrast with the spectral changes observed upon titrations of the protein with heterocyclic compounds (type II P450 responses,^32^ a red shift in the Soret band maximum, occurring due to replacement of the water molecule in the iron coordination sphere by a more basic nitrogen atom in the heterocyclic ring). All of them produced very similar *K*_d_ values in the low nanomolar range (1–7 nM), with the titration maximum being reached at approximately equimolar ratio protein/ligand (Figure 4C; the corresponding titration curves for the other tested heterocyclic compounds can be found in Figure S2).

### Inhibition of CYP51 Catalysis with Clinical Azoles

**Figure 5:**
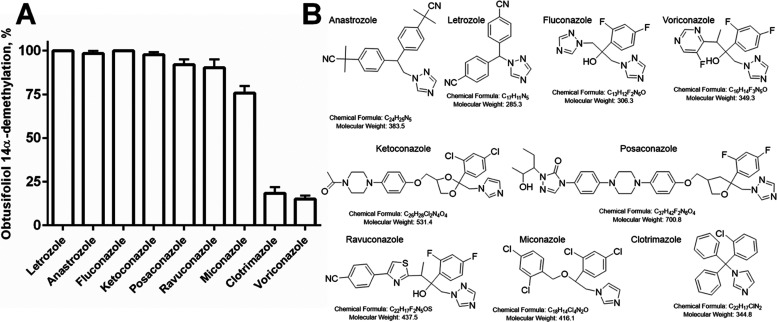
Clinical drugs. (A) Inhibition of *A. castellanii* CYP51 catalysis by commercial azole-based drugs in a 1 h reaction at the drug/enzyme molar ratio 3/1 (final concentrations 1.5/0.5 μM). The concentration of obtusifoliol was 50 μM. The experiments were performed in triplicate; the results are presented as mean ± SD. (B) Structural formulas.

First, a set of commercially available azole-based clinical drugs were screened against *A. castellanii* CYP51 to evaluate the range of their inhibitory potencies and the overall susceptibility of the enzyme as a target. In these experiments, we used a 3-fold molar excess of an inhibitor over P450 and a 1 h reaction (Figure 5). These conditions have been previously established as time-efficient and informative, allowing for the rapid elimination of less potent compounds and the selection of functionally irreversible (ideally stoichiometric) inhibitors.^33−35^ Among the drugs tested, voriconazole and clotrimazole were the strongest inhibitors, preventing 14α-demethylation of 85 and 82% of the substrate in the reaction mixture, respectively, under these conditions. Miconazole inhibited conversion of obtusifoliol by 25%. The effects of the other azole drugs were negligible, indicating that they were easily outcompeted by the substrate over time.

### Inhibition of CYP51 Catalysis with Chemically Synthesized Experimental Compounds

**Figure 6:**
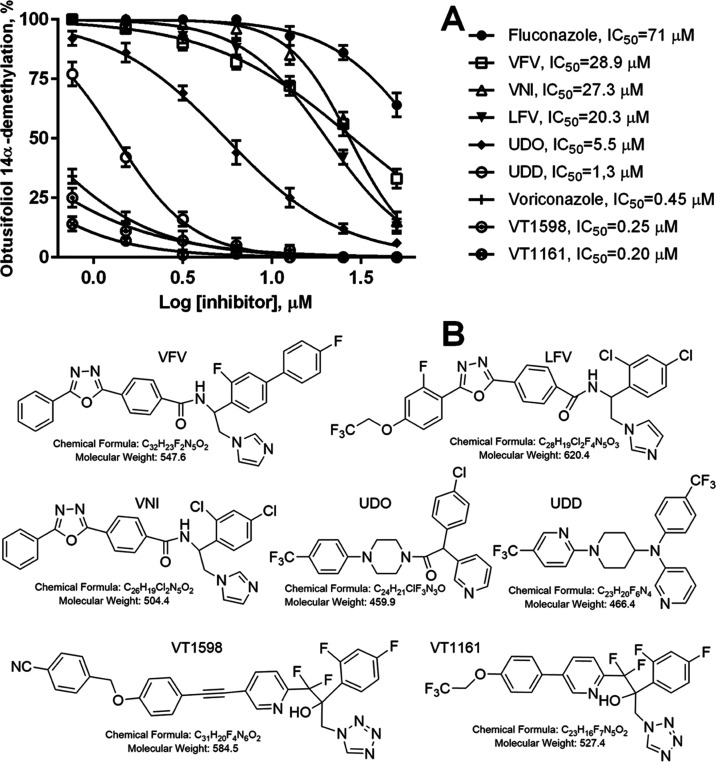
Experimental CYP51 inhibitors. (A) Dose-dependent effects of imidazole-, pyridine-, and tetrazole-based CYP51 inhibitory scaffolds on the activity of the *A. castellanii* ortholog, 1 h reaction. Fluconazole and voriconazole were used as controls. P450 concentration was 0.5 μM; obtusifoliol concentration was 50 μM. The experiments were performed in triplicate; the results are presented as mean ± SD (B) structural formulas.

Voriconazole and fluconazole were chosen for further experiments as positive and negative controls, respectively. Both of these antifungals were reported to have some effectiveness in the treatment of human infections by *Acanthamoeba* (e.g., fluconazole,^36−40^ voriconazole^11,41,42^), suggesting that the chemical scaffolds with the 1 h IC_50_ values lower than that of fluconazole (71 μM) can all be considered as potential leads for further drug design. Within the range (Figure 6) were our synthesized imidazole-based VFV (29 μM), VNI (27 μM), and LFV (20 μM), the structures previously identified and further developed as inhibitors of protozoan or fungal CYP51 orthologs.^43−45^ They were followed by the pyridine derivatives UDO (5.5 μM) and UDD (1.3 μM), the compounds from the Drugs for Neglected Diseases Initiative (DNDi), once considered as drug candidates for Chagas disease.^46^ The best values (<IC_50_ of voriconazole, 0.45 μM) were obtained for the tetrazoles VT1598 (0.25 μM) and VT1161 (0.20 μM), both potent inhibitors of *Candida albicans* and *A. fumigatus* CYP51s.^47,48^ VT1598 is currently in clinical trials, and VT1161 was recently approved by the FDA as a clinical antifungal.^49−51^

### Inhibition of *Acanthamoeba* Cell Growth

Compounds known to have good blood–brain permeability and low toxicity in animal studies, including VNI and VFV,^45^ were selected for cellular experiments (Table 2). Overall, the results of their inhibition of *A. castellanii* CYP51 in our reconstituted 1 h enzyme reactions correlated with their activities against *A. castellanii* cells. The effect of VT1161 (minimal inhibitory concentration (MIC) = 0.057 μM) was the strongest, followed by VT1598 (0.077 μM), and then by voriconazole (0.09 μM). Relatively weak as an *A. castellanii* CYP51 inhibitor, VNI yielded a MIC of 4 μM, while the MIC of fluconazole was above the limit of the cellular assay (>64 μM). The activities of the compounds against *A. polyphaga* were generally substantially lower. This result was quite unexpected, considering the high sequence identity of their CYP51 enzymes (>99%). VT1161 was the only exception, highly active against both species, indicating this new drug’s potential to work as an assassin of various pathogenic Amoebozoa.

**Table 2:** MIC of Azoles against Amoebae Cells

	MIC, μg/mL	
azole	A. castellanii	A. polyphaga
fluconazole	>64	>64
voriconazole	0.03 [0.090 μM]	0.25 [0.75 μM]
VNI	8 [4 μM]	>64
VFV	>64	>64
VT1598	0.045 [0.077 μM]	0.25 [0.425 μM]
VT1161	0.03 [0.057 μM]	0.06 [0.114 μM]

One possible explanation for the observed interspecies differences in cellular activities of the other tested CYP51 inhibitors might be that VT1161, VT1598, voriconazole, and perhaps VNI target another *A. castellanii* P450 and this P450 enzyme is also important for the lifecycle of the pathogen. Our bioinformatic analysis of the *A. castellanii* genome reveals the presence of 27 putative P450 enzymes, identified by the presence of consensus motifs, such as the ExxR in the K helix, the motif around the cysteine, which forms the fifth axial ligand to the heme iron, and the motif around the conserved threonine residue in the I helix, which is involved in oxygen activation. Examination of the *A. castellanii* P450s revealed two P450 enzymes, CYP51 (sterol 14α-demethylase) described herein and CYP710D2 (sterol 22-desaturase), that are involved in the *A. castellanii* sterol biosynthetic pathway. The remaining 25 P450s are “orphans”, having no known function that can be inferred by comparative sequence identity analysis or by published experimental work to date. The designations of the remaining 25 putative *A. castellanii* P450s, as annotated, are CYP745D1, 5645A1, 5645A2, 5645B1, 5645C1, 5645D1, 5646A1, 5646B1, 5647A1, 5647A2, 5648A1v1, 5648A1v2, 5649A1, 5649B1, 5650A1, 5651A1, 5652A1, 5653A1, 5654A1, 5655A1, 5656A1, 5657A1, 5658A1, 5659A1, and 6008A1. CYP6008A1 is a predicted fusion protein consisting of a P450 domain and a peroxidase domain.

### Crystallization Construct

For crystallization, we used the *A. castellanii* CYP51 construct with the hydrophobic N-terminal membrane anchor sequence deleted up to the proline-rich region^52^ (Pro44 in *A. castellanii* (Figure S1, marked blue)). This truncation apparently not only facilitates CYP51 crystallization but also prevents crystallographic artifacts because the position of the transmembrane helix in the crystals cannot possibly reflect its actual arrangement in the endoplasmic reticulum membrane. Identically to full-length *A. castellanii* CYP51, the truncated protein was purified in the low-spin form with the Soret band max at 418 nm, the spectrophotometric index OD_418_/OD_278_ was 1.7, and no admixture of inactive P420 form was detected in the reduced carbon monoxide binding spectrum. The truncated protein was fully catalytically active, as are many similarly truncated microsomal P450s,^53^ and more stable. Moreover, the addition of the substrate resulted in an almost complete (>95%) low-to high-spin state transition in the heme iron (Figure S3, see Figure 3 for comparison), a feature rare among the CYP51 enzymes.^54^

### Crystallographic Analysis of *A. castellanii* CYP51 in the Absence of an Inhibitor

In the absence of an inhibitor, *A. castellanii* CYP51 crystallized in the *P*2_1_2_1_2_1_ space group, and the structure was refined to 1.95 Å, with the *R*_work_/*R*_free_ = 0.19/0.22, the average *B*-factor 25.3 Å^2^ (PDB code 7UWP) (Table S1). The asymmetric unit consisted of two protein molecules in a dimeric arrangement, with the N-terminus of one molecule (starting from Leu43) interacting with the β2-sheet of another in such a way that each protein globule shields the distal surface of the other in the hydrophobic area commonly known as “the substrate entrance channel” (helices A′ and F″ and the β4 hairpin), pushing helix A′ ∼ 10 Å up and toward helix F″ (Figure S4A,B). As a result of these rearrangements (most likely occurring upon crystallization), the substrate entrance closes completely (Figure S4C), an event unusual for CYP51 enzymes of resolved structure although possibly common among the *Acanthamoeba* orthologs, because they carry two sequential glycine residues in the short (-AG_62_G_63_P-) A/A’ loop (Figure S1, marked in yellow), and glycines increase the P450 backbone plasticity.

**Figure 7:**
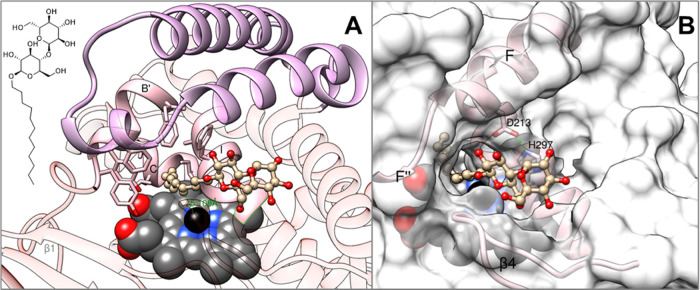
Binding mode of *n*-dodecyl-β-d-maltoside. (7UWP). (A) Inside the active site. The heme-bound water molecule is shown as a black sphere. The detergent (tan carbons) is in ball and stick representation, and the residues within 4.5 Å from its fatty acid tail are shown as sticks. The corresponding secondary structural elements are pink, and the rest of the ribbon is semitransparent. The FG arm (195–256) is plum. Inset: the detergent structural formula. (B) Outside the protein globule. Surface representation. The detergent-formed channel between helices F, I, and the β4 hairpin is seen through the semitransparent surface. The proton relay salt bridge (D213-H297) is on the right.

As expected, there was a water molecule bound at the sixth coordinate position of the heme iron in each protein molecule. The distance to the iron atom, however, was shorter, 2.5 Å vs, e.g., 2.9 Å in 3G1Q,^55^ probably because of the presence of the detergent (*n*-dodecyl-β-d-maltoside) (Figure S4). The hydrophobic fatty acid (dodecyl-) tail of the detergent molecule is positioned deep inside the CYP51 active site, with its C5 atom lying 5 Å above the iron-coordinated water. Above the heme plane, the tail of the detergent is surrounded by the residues from the B′ helix (Tyr114, Phe116, Ile120, Phe121), B′C loop (Val126, Tyr127), and helices C (Leu138, Ile141) and I (Ile290, Phe293, Ala294) (Figure 7A). In contrast, the polar (glycoside-) head is exposed to the bulk solvent protruding through the protein surface by ∼9 Å. Interestingly, it escapes the active site by forming a new channel, an opening between the tip of the β4 hairpin and helices F and I, directly above the putative proton delivery route,^54^ though the conserved salt bridge between Asp231 and His297 remains closed, as it normally does in CYP51 molecules in the absence of the substrate (Figure 7B). This pattern is similar to what was observed with another detergent, Anapoe-X-114 (23-(4-(2,4,4-trimethylpentan-2-yl)phenoxy)–3,6,9,12,15,18,21-heptaoxatricosan-1-ol), which we previously found cocrystallized in the structure of the soluble CYP51 from *Methylococcus capsulatus* (6MCW)^56^ (Figure S4B). Formation of this “special” channel around the detergents supports the theory that functionally essential conformational dynamics of CYP51s in vivo involves large-scale movements of the flexible (elastic) FG arm that result in a wide opening of the cleft bordered on the opposite side by helices A′, I and the β4 hairpin and lined by the steady β1-sheet floor.^31^ Typically, following molecular recognition, when a high-affinity ligand enters the CYP51 active site, the entrance closes partially (e.g., for most inhibitors^24^) or completely (e.g., for substrates^54,60^), and a long arm of an inhibitory molecule (which are much less polar than the polar heads of the detergents) is found trapped within the hydrophobic channel formed between helices A′ and F″ and the β4 hairpin. (It is often called the substrate entrance channel and is most likely involved in the passage of the substrate molecules toward the place of catalysis).

**Figure 8:**
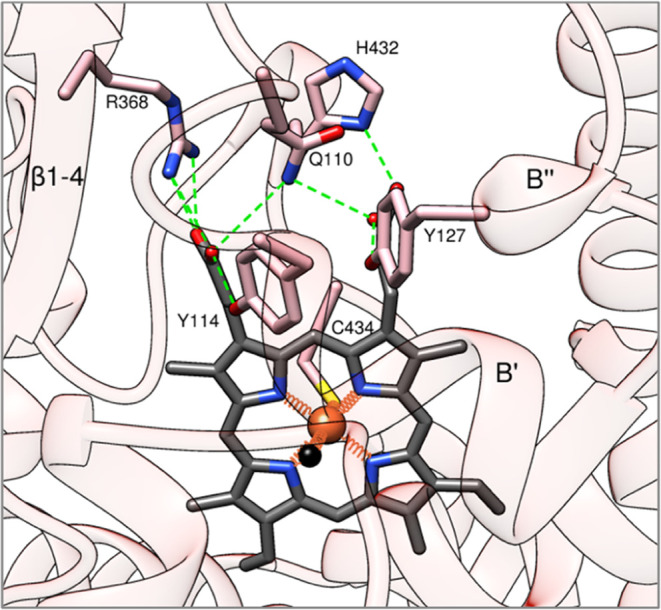
Heme surrounding in *A. castellanii* CYP51 (7UWP). Six protein residues forming interactions with the heme are labeled. Hydrogen bonds are shown as dashed green lines and iron coordination bonds are presented as orange springs. The secondary structural elements are seen as semitransparent ribbons.

Despite the nuances described above, the overall *A. castellanii* CYP51 structure is similar to the structures of other CYP51 orthologs that we have previously determined (e.g., the RMS deviations for all Cα atoms between the *A. castellanii* (7UWP) and *M. capsulatus* (6MCW)/water-bound *T. brucei* (3G1Q) structures are only 1.4/1.6 Å). The length and position of the secondary structural elements downstream of the A′ helix are well preserved (as seen in Figure S4B). Cys434 serves as the fifth (proximal) axial ligand to the heme iron. The heme support from the rest of the protein molecule is provided by five residues that form H-bonds with the A- and D-ring propionates (Figure 8). Tyr114 (B′ helix), Arg368 (β1–4 strand), and His432 (the heme bulge) are invariant throughout the whole CYP51 family. Tyr127 (the helical turn (B″) within the B′C loop) is conserved in CYP51 enzymes of animals, fungi, and protozoa, while CYP51s of plants and green algae always have Phe in this position. The side chain nitrogen of Gln110 (loop BB′) interacts with the propionate oxygens on both porphyrin rings. This interaction is so far unique for the *A. castellanii* CYP51 structure, but it is likely to be found in other *Acanthamoebae* as well as in algae and plants, where this glutamine residue is conserved (Figure S1, pink). Evidence has been presented that H-bonds with the porphyrin ring influence P450 redox potential,^57,58^ a feature deserving further investigation because, by influencing the enzyme catalysis, it might be connected with its susceptibility to inhibition.

### Aggregation State of *A. castellanii* CYP51 in Solution

**Figure 9:**
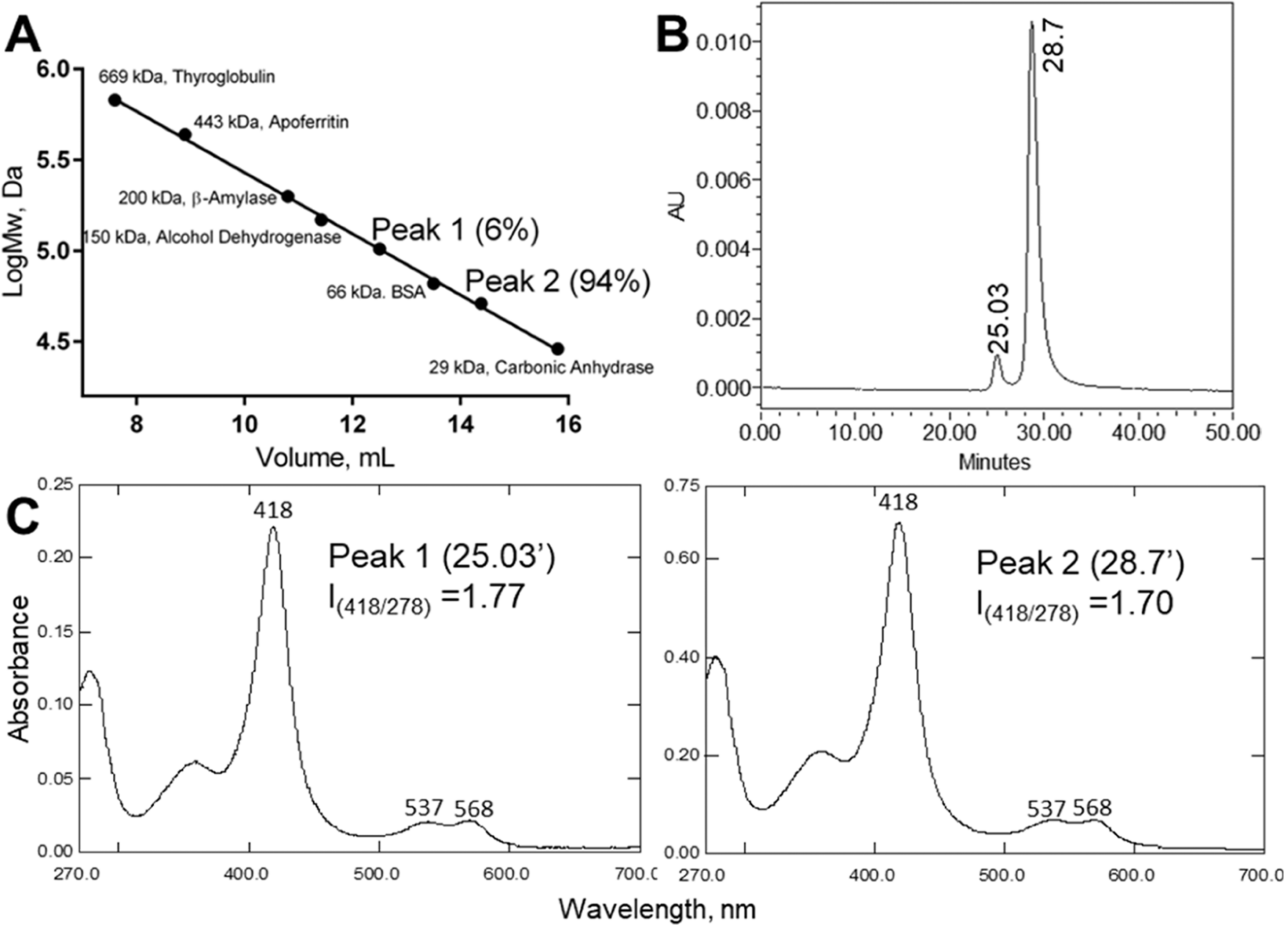
Size-exclusion chromatography on Superdex 200 increase 10/300 GL. (A) Column calibration in PBS and 0.5 mL/min flow rate. (B) The HPLC profile of the *A. castellanii* CYP51 sample. (C) Absolute absorption spectra of peaks 1 (dimer) and 2 (monomer).

We analyzed the aggregation state of our crystallization construct in solution because, in the absence of an inhibitor, it is crystallized as a dimer (Figure S4A). Our results indicated that in PBS (phosphate buffer saline, pH 7.4), the *A. castellanii* CYP51 protein was mainly monomeric (94%), though only with a small (6%) admixture of a dimer present (Figure 9A,B). Both chromatographic peaks (monomer and dimer) were isolated and produced normal absolute absorption spectra, typical for a low-spin water-bound P450, as indicated by the position of the Soret band maximum (418 nm) and almost equal absorbance of the β- and α-bands (at 537 and 568 nm, respectively) (Figure 9C). Since our purified monomeric CYP51 protein was fully active, we conclude that the dimerization found in the crystals is an artifact of crystallization and is not required at all for *A. castellanii* CYP51 enzyme function and catalysis.

### Crystallographic Analysis of VT1161-Bound *A. castellanii* CYP51

VT1161 was selected for cocrystallization because, in this work, it was found to be the most potent inhibitor of *Acanthamoeba* sp. Additionally, it has been previously shown that VT1161 does not bind to human CYP51,^59^ indicating the desired selectivity. It is noteworthy, however, that even being the strongest among the tested compounds, VT1161 did not act as a stoichiometric inhibitor of *A. castellanii* CYP51. Figure 6 shows that a 1.5-fold molar excess of VT1161 over the enzyme (the lowest inhibitory concentration used in the assay) resulted in ∼15% of the substrate conversion into the product. VT1161-bound *A. castellanii* CYP51 crystallized in the P1 space group, and the structure was refined to 2.28 Å, with the *R*_work_/*R*_free_ = 0.21/0.22 and the average *B*-factor 46.0 Å^2^, the PDB code 8EKT (Table S1). The asymmetric unit consisted of six very similar protein molecules, with the average RMSD between their Cα atoms being 0.22 Å. Each protein molecule contained one molecule of the inhibitor bound in essentially the same conformation, except for some rotations of the very distal portion of its long arm (trifluoroethoxy group). There was no protein dimerization in this structure, and the mutual orientations of the polypeptide chains differed from those in 7UWP. In addition, Lys77 (the final residue in helix A in 7UWP) was the first N-terminal amino acid seen in the electron density map. The density for the preceding 34-residue fragment (or even space for some density) was missing. Sodium dodecyl sulfate–polyacrylamide gel electrophoresis (SDS–PAGE) analysis of the sample used for crystallization versus the sample from dissolved crystals confirmed proteolytic degradation of the N-terminal fragment during the crystallization process.

**Figure 10:**
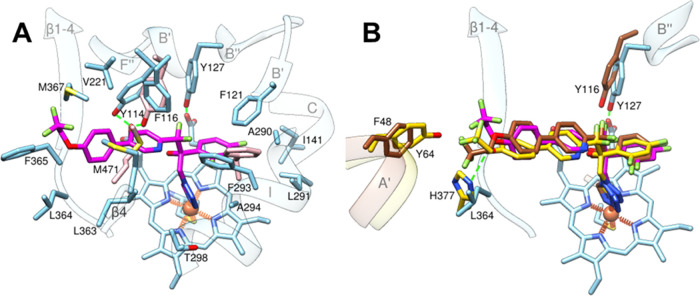
VT1161 binding mode (8EKT). (A) Inside the active site of *A. castellanii* CYP51. The inhibitor (magenta carbons), the heme, and amino acid residues within 4.5 Å (blue carbons) are shown as sticks, and the corresponding secondary structural elements are seen as semitransparent ribbons. Y114, F293, and M471 (pink carbons) are from the superimposed inhibitor-free structure. (B) Superimposition of 8EKT with VT1161-bound T. cruzi (brown, 5AJR) and *C. albicans* (yellow, 5TZ1) CYP51s. F48/Y64 are from helix A′ (F60 in *A. castellanii*); Y116 in complex with VT1161 in *T. cruzi* CYP51 loses its H-bond with the heme propionate (Y127 in *A. castellanii*). H377 in *C. albicans* forms a H-bond with the trifluoroethoxy oxygen of the inhibitor (L364 in *A. castellanii*). Hydrogen bonds are shown as dashed green lines and iron coordination bonds are presented as orange springs. The secondary structural elements are seen as semitransparent ribbons.

Overall, the binding of VT1161 did not cause any substantial rearrangements in the protein backbone. In the middle of the I helix, the turn carrying the conserved P450 threonine involved in oxygen activation (Thr298 in *A. castellanii*) was shifted ∼1 Å toward the heme iron atom. The tip of the β-hairpin moved 1.6 Å toward helix F. Some differences are also seen in the positions of loops GH and H, the areas known to be the most flexible or even disordered in P450s. The average RMSD between the Cα atoms in the 8EKT and 7UWP structures is 0.65 Å. Within the active site, the N4 nitrogen of the VT1161 tetrazole ring forms a 2.2 Å-long coordination bond with the heme iron. The β-phenyl ring lies deeper inside, reaching helix C (Ile141), while the long arm is directed toward the distal P450 surface, occupying the “traditional CYP51 substrate entrance“ space (where the misplaced helix A′ lies in 7UWP). Due to the lack of helices A and A′ in the structure, the trifluoroethoxy group of VT1161 is partially exposed to the solvent and has a higher *B*-factor (up to 90 A^2^ vs 8–10 A^2^ for the tetrazole and β-phenyl rings inside the active site). At van der Waals distances (<4.6 Å, Figure 10A), the inhibitor is contacted by 16 mainly hydrophobic amino acid residues. Tyr114, Phe116, Phe121, and Tyr127 are from the B′ helix-B′C loop area; Tyr141 is from helix C, and Val221 from helix F′. Ala290, Leu291, Phe293, Ala294, and Thr298 are from the middle of helix I. Leu363, Leu364, Phe365, and Met367 are form strand β1–4 and the preceding loop (SRS4, shown in Figure 1), Met471 is from the β4 hairpin. The positions of most of these residues are similar to those in the structure without a bound inhibitor, except for Phe293 and Met471, which adopt different conformations, and Tyr114, whose side chain ring rotates ∼ 90° yet does not lose its H-bond with the heme. Thus, the strong inhibitory effect of VT1161 on the activity of *A. castellanii* CYP51 does not appear to be a result of some inhibitor-induced large-scale conformational change in the enzyme. We surmise that, instead, multiple interactions between VT1161 and the protein moiety impede the conformational rearrangements that are required for catalysis (e.g., acquiring the substrate,^54^ redox partner recognition^60^).

This view is supported by the high similarity of the VT1161 binding mode in all of the three costructures with the CYP51 enzymes that we have determined (Figure 10B). Despite the low amino acid sequence identity between the CYP51 orthologs, most VT1161 contact residues (Table S2) coincide in the multiple sequence alignment (as can also be seen in Figure S1), even though they are often phyla-specific. For example, the phylum-specific His377 in the *C. albicans* structure forms a H-bond with VT1161. The 2.8 Å hydrogen bond must be stronger than the hydrophobic interactions with the corresponding Leu364 seen in *A. castellanii*. This result is consistent with the higher potency of the inhibitor toward *C. albicans* CYP51,^47^ and conservation of this histidine residue across fungal species provides a structural basis for the broad-spectrum antifungal activity of the compound.^48^ In this connection, the high potency of VT1161 toward *T. cruzi* CYP51 (∼95% inhibition at a 2-fold molar excess over the enzyme^61^) can be explained by the Tyr116 rearrangement. In complex with the inhibitor, the oxygen atom of the Tyr116 side chain is found 4.1 Å away from the heme propionate, and as we observed previously, loss of the H-bonds between these tyrosines and the heme prosthetic group often accompanies a strong inhibitory effect of a compound, even when the number of its contacts with the protein is lower.^35,62^ Finally, based on this structural comparison, there must be at least one more VT1161-contacting residue in *A. castellanii* CYP51 (Phe60 from helix A′, which is missing in the 8EKT structure).

### Structural Explanation for the Potency of Voriconazole

**Figure 11:**
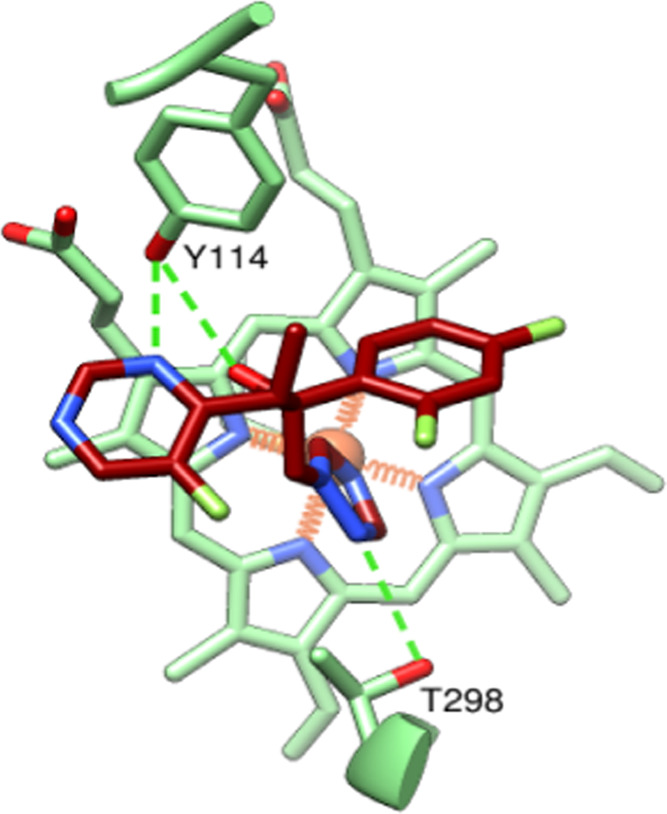
Voriconazole docked in the 7UWP structure of *A. castellanii* CYP51. The inhibitor (dark red carbons), the heme, Y114, and T298 (light green carbons) are shown as sticks and possible H-bonds are depicted as dashed green lines.

The antifungal drugs fluconazole and voriconazole are of the same structural scaffold. They differ only in the composition of one ring, 1,2,4-triazolyl- versus 5-fluoro-4-pyrimidinyl- (Figure 5B), molecular volumes 348 and 399 Å^3^, respectively. Yet, the differences in their potencies to inhibit *A. castellanii* CYP51 are distinctly profound (Figure 6A, the 1 h IC_50_ of voriconazole is ∼160-fold lower). Docking of voriconazole in the *A. castellanii* CYP51 structure suggests that the pyrimidine ring nitrogen (N3) of voriconazole binds the hydroxyl of Tyr114 (Figure 11).

This would be somewhat similar to what we have previously found in the structure of voriconazole-bound CYP51 from *A. fumigatus* (4UYM), a CYP51B enzyme that is also strongly inhibited by voriconazole but not by fluconazole.^29^ There is also a possibility that voriconazole can constrict *A. castellanii* CYP51 with two further H-bonds (between its 2-OH group and Tyr114 and between the heme-coordinated triazole ring (N2) and the hydroxyl oxygen of Thr298). Crystallographic interrogation of voriconazole binding to *A. castellanii* CYP51 is currently underway in our laboratory.

### Structural Basis for the Strict Substrate Preferences

**Figure 12:**
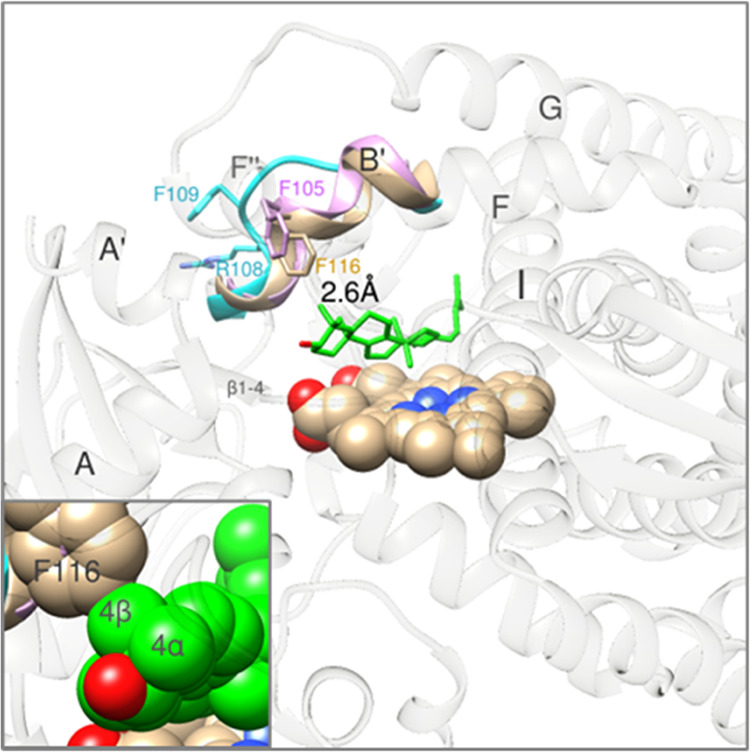
Substrate-gating residue in the B′ helix. *A. castellanii* CYP51 (8EKT, tan) is superimposed with the structures of T. brucei (plum, 3G1Q), *N. fowleri* (cyan, 7RTQ), and lanosterol (green)-bound human (semitransparent gray, 6UEZ) orthologs. Membrane-oriented side of the molecule. Inset: Enlarged view of *A. castellanii* F116 and lanosterol in sphere representation; the C4 atom α and β methyl groups (C28 and C29 carbons) are marked. The clash between the 4β methyl group of lanosterol and F116 indicates that in *A. castellanii* CYP51, a structural rearrangement in the B′ helix is required for this substrate to reach the catalytic site.

Comparative structural analysis of CYP51s from *A. castellanii*, *T. brucei* (another obtusifoliol-specific sterol 14α-demethylase^63^), and *N. fowleri*(31) provides an explanation for the observed variations in the substrate requirements of these enzymes, each having plant-like Phe in the B′ helix (Figure 12). In *A. castellanii*, as well as in *T. brucei*, the aromatic ring of this residue is found positioned inside the substrate-binding cavity, supporting its role as a gating residue^64^ and most likely guiding the substrate toward the site of catalysis. Moreover, in the structure of I105F *T. cruzi* CYP51, in complex with obtusifoliol (6FMO), Phe105 lies within 4.1 Å of the C4 atom of the sterol molecule facing its β-surface, right where the second methyl group (C29, or 4β) decorates lanosterol. The position of lanosterol in the human CYP51 structure in Figure 12 is the same as that of obtusifoliol in 6FMO,^54^ showing that for lanosterol to reach the catalytic site, an elastic movement of helix-B′ in *A. castellanii* CYP51 must be required. Based on the results of our molecular dynamics simulations, the B′ helix in CYP51s is overall highly rigid,^64^ but the enzyme from *N. fowleri* appears to represent an exception. In its B′ helix, Phe109 is flipped and positioned above the surface of the protein. Most likely, this happens because of the positively charged guanidinium group of neighboring Arg108. Having a high propensity to be solvent accessible, it drags Phe109 to the surface, weakening the main chain interactions and thus making this structural segment more flexible. A positively charged amino acid preceding this phyla-specific (Phe vs Leu) residue in the B′ helix is overall rather rare in CYP51 sequences. It is, however, conserved in vertebrates (predominantly Arg, sometimes Lys). This includes human CYP51, whose resistance to inhibition is quite notorious.^65−67^ We propose that restricted mobility of Phe116 in *A. castellanii* CYP51 not only defines (restricts) its substrate preferences but also suggests that functionally irreversible stoichiometric inhibitors (e.g., like VNI for *T. brucei* CYP51^55^) can be designed to target this sterol 14α-demethylase.

## Conclusions

Although the final sterol products of the pathway in acanthamoebas are ergosterol-like, as they are in fungi and Trypanosomatida, the results of this work support the plant-like (squalene to cycloartenol to obtusifoliol) portion of the ergosterol pathway in *Acanthamoeba*(19) and thus the notion that ”protozoa may be a polyphyletic group, some of which evolved from an algal ancestry, while others did not.”^23^ The GCMS numbers/data reported by Tomson et al.^20^ are insufficient to indicate an animal-like pathway because lanosterol and obtusifoliol have the same molecular weight (see Figure 3). The residual ability of the *A. castellanii* CYP51 enzyme to 14α-demethylate lanosterol (even though much slower than obtusifoliol) supports the involvement of conformational rearrangements in the substrate binding process. Structural analyses of CYP51 orthologs across phylogeny advocate that the lack of correlation between the spectrally determined apparent binding affinities of the heme iron-coordinating ligands and their long-term inhibitory effects on the enzymatic activity,^33,34,45,68^ as observed again here for *A. castellanii* CYP51, indicate that while the spectral *K*_d_ values reflect the fact (or perhaps easiness) of the initial coordination of a heterocyclic warhead to the heme iron, they should not be used solely to judge inhibitory potencies. We surmise that the most potent heme-coordinating CYP51 inhibitors are those that “freeze” the enzyme in its half-open (ligand-free-like^24^) conformation. This can be done via multiple interactions with the protein moiety, preventing the FG arm from further opening, which is required for the substrate to enter the active site. The structures determined here can facilitate the rational design of new, potent, and selective inhibitors of *A. castellanii* CYP51, ideally stoichiometric and functionally irreversible,^34,45,68^ while both voriconazole and VT1161 can effectively serve as *Acanthamoeba* killers, with the advantages being that they are already in clinical use, and both penetrate the blood–brain barrier. Considering that current treatments of *Acanthamoeba* keratitis take several months, the disinfectants (like biguanide and chlorhexidine) are only used topically, and oral miltefosine is toxic, and even at highly aggressive doses, its concentration in cerebrospinal fluid remains negligible,^69^ systematic use of potent CYP51 inhibitors (particularly those with good blood–brain barrier permeability) as combination therapy with the currently available treatment regimens should be clinically significant, with the benefits including lower toxicity and faster recovery.

## Experimental Section

### Reagents

The azole-based drugs ketoconazole, posaconazole, voriconazole, ravuconazole, clotrimazole, miconazole, letrozole, and anastrozole were purchased from Santa Cruz Biotechnology (Dallas, TX), and fluconazole was from ICN Biomedicals. VNI and VFV were synthesized by the Chemical Synthesis Core Facility (Vanderbilt Institute of Chemical Biology).^70^ The synthesis of their analogue LFV was described previously.^68^ The purity of the compounds was >95%. The pyridine derivatives UDO and UDD were from DNDi;^46^ the tetrazole-based compounds VT1161^47^ and VT1598^48^ were from Mycovia Pharmaceuticals (Durham, NC). Hydroxypropyl-β-cyclodextrin (HPCD) was purchased from Cyclodextrin Technology Development (Gainesville, FL). Q- and CM-Sepharose resins were from GE Healthcare, and Ni^2+^-nitrilotriacetate (NTA) agarose was from Qiagen. The synthesis of the CYP51 reaction product (3β,5α)-4,4-dimethyl-cholesta-8,14,24-trien-3-ol (dihydro-FF-MAS) was reported elsewhere.^71^ All compounds were >95% pure by high-performance liquid chromatography (HPLC).

### CYP51 Sequences

Blast search analysis was carried out using the NCBI database (https://blast.ncbi.nlm.nih.gov/Blast.cgi). Multiple sequence alignment was performed in Clustal Omega and analyzed in GenDoc. The accession numbers of the proteins are as follows: *A. castellanii*: XP_004334294.1; *A. polyphaga*: AMN14212.1; *Galdieria partita*: GJQ09410.1; *Galdieria sulphuraria*: XP_005708760.1; *G. theta*: XP_005826589.1; *T. trahens*: XP_013758049.1; *Chlorella sorokiniana*: PRW59933.1; *Prototheca miyajii*: QQD79796.1; *Ostreococcus lucimarinus*: XP_001420297; *Volvox reticuliferus*: GIL72098; *Gonium pectorale*: KXZ44315; *Geum rivale*: CCH26420.1; *Papaver californicum*: KAI3993583; *Vitis vinifera*: XP_002271942; *Linum tenue*: CAI0556290; *N. fowleri*: KAF0972476.1; *Strongylocentrotus purpuratus* [sea urchin]: NP_001001906.1; *Salmo salar* [salmon]: XP_013997770; *Bufo bufo* [toad]: XP_040287086; *Tyto alba* [owl]: XP_032847495; *Homo sapiens* [human]: NP_000777.1; *T. brucei*: XP_828695.1; *T. cruzi* strain Y 51A: AFW98339.1; *T. cruzi* strain Y 51B: AFW98340.1; *Cryptococcus neoformans:* XP_566464.1; *Saccharomyces cerevisiae*: AJU25839; *A. fumigatus* 51B: XP_749134.1; *Neosartorya fischeri* 51B: XP_001261295.1; *Fusarium graminearum* 51B: ACL93392.1; *Ophiocordyceps sinensis* 51B: EQK98774.1; *A. fumigatus* 51A: AAF32372.1; *N. fischeri* 51A:; *F. graminearum* 51A: ACL93390.1; *O. sinensis* 51A: EQL02138.1; *Daldinia loculata* 51A: XP_049162104.1; and *Metarhizium brunneum* 51A: QLI68756.1.

### *A. castellanii* CYP51 Gene and Expression Vector Construction

The *A. castellaniiCYP*51 gene, codon-optimized for bacterial expression, was synthesized by Eurofins MWG Operon (Ebersberg, Germany), incorporating an *Nde*I restriction site at the 5′-end and a *Hin*dIII restriction site at the 3′-end of the gene cloned into the pUC57 plasmid. The second amino acid (Val) was changed to Ala (–GGC−) to enhance protein expression, and a six-histidine extension (–CATCATCACCATCATCAC) was inserted before the stop codon (–TAA) to facilitate purification by NTA agarose affinity chromatography. This gene encoding the full-length CYP51 protein, 492 amino acid residues plus the (His)_6_-tag, ∼56 kDa, calculated pI 6.6 (Antheprot), was used for functional studies, including ligand binding, enzymatic activity, and inhibition assays. For crystallization purposes, the protein was truncated (to 460 amino acid residues, ∼52 kDa, calculated pI 7.0) as follows: the 43-amino acid sequence at the N-terminus (up to the conserved CYP51 proline, P44 in *A. castellanii* CYP51) was replaced with the 11-amino acid sequence fragment MAKKTSSKGKL-(ATGGCTAAGAAAACGAGCTCTAAA GGGAAGCTC−) as described previously for *A. fumigatus* CYP51.^29^ For protein expression, both the full-length and truncated *CYP*51 genes were excised by *Nde*I/*Hin*dIII restriction digestion followed by cloning into the pCW expression vector using NEB T4 DNA ligase. Gene integrity was confirmed by DNA sequencing.

### Protein Expression and Purification

*A. castellanii* CYP51 was expressed in *Escherichia coli* HMS-174 (DE3) (Novagen) as described previously for *A. fumigatus* CYP51^29^ and then purified to electrophoretic homogeneity in three steps, including affinity chromatography (Ni^2+^–NTA agarose), anion exchange chromatography on Q-Sepharose, and cation exchange chromatography on CM-Sepharose. In general, we followed the described procedure,^29^ except that the (NTA–) bound protein was eluted in 50 mM potassium phosphate buffer, pH 7.8, containing 100 mM NaCl, 10% (v/v) glycerol, 0.1 mM EDTA, and 120 mM imidazole. The fractions with a spectrophotometric index (*A*_425_/*A*_280_) ≥ 1.1 were pooled and concentrated using an Amicon Ultra 50 K (Millipore) concentration device to a volume of 5 mL, then diluted 5-fold with 50 mM potassium phosphate buffer, pH 7.8, containing 10% (v/v) glycerol and 0.1 mM EDTA and applied to a Q-Sepharose column equilibrated with the same buffer. The flowthrough fractions with a spectrophotometric index (*A*_418_/*A*_280_) ≥ 1.5 were concentrated, diluted 5-fold with 20 mM potassium phosphate buffer, pH 6.8, containing 20 mM NaCl, 10% (v/v) glycerol, and 0.1 mM EDTA and applied to a CM-Sepharose column. The column was washed with the same buffer with a linear gradient of NaCl (20–100 mM), and the protein was eluted with 20 mM K-phosphate buffer, pH 7.4, containing 150 mM NaCl, 10% (v/v) glycerol, and 0.1 mM EDTA. The procedure was the same for the full-length and truncated *A. castellanii* CYP51 constructs. The functionality of the truncated enzyme was verified prior to crystallization.

Recombinant rat NADPH-cytochrome P450 reductase (CPR) was also expressed in *E. coli* and purified as described elsewhere.^72^

### UV–Visible Spectroscopy

UV–visible absorption spectra (270–700 nm) were recorded at 22 °C using a dual-beam Shimadzu UV-2600i spectrophotometer. The P450 concentration was determined from the Soret band absorbance in the absolute spectrum using the extinction coefficient of 117 mM^–1^ cm^–1^ for the low-spin oxidized (ferric) form of the protein or 91 mM ^–1^ cm^–1^ for the reduced carbon monoxide (ferrous-CO) complex in the difference spectra.^73^ The spin state of the P450 samples was estimated from the absolute spectra as the ratio (Δ*A*_393–470_/Δ*A*_417–470_), with values of 0.35 and 2.0 corresponding to 100% low- and 100% high-spin iron, respectively. Spectral titrations with sterol substrates were carried out at 2 μM P450 concentration in 50 mM potassium phosphate buffer, pH 7.4, containing 100 mM NaCl and 0.1 mM EDTA, in 1 cm optical path length cuvettes. Sterol binding was monitored as a blue shift in the P450 Soret band maximum.^32^ Aliquots of 0.5 mM stock solutions of sterols in 45% (w/v) HPCD^63^ were added to the sample cuvette in the concentration range 0.25–5 μM, with each titration step being 0.25 μM. At each step, the corresponding volume of 45% (w/v) HPCD was added to the reference cuvette. Titration with heterocyclic compounds was carried out at 0.5 μM P450 concentration in 5 cm optical path length cuvettes. The binding of these ligands was monitored as a red shift in the P450 Soret band maximum.^32^ Aliquots of 0.1 mM compounds dissolved in DMSO were added to the sample cuvette in the concentration range 0.1–1.0 (1.5) μM, with each titration step being 0.1 μM. At each step, the corresponding volume of DMSO was added to the reference cuvette. The apparent spectral dissociation constants (*K*_d_) were calculated in GraphPad Prism software by fitting the data for the ligand-induced absorbance changes in the difference spectra Δ(*A*_max_*– A*_min_) versus ligand concentration to the quadratic equation Δ*A* = (Δ*A*_max_/2E)((L + E + *K*_d_) – ((L + E + *K*_d_)^2^ – 4LE)^0.5^), where [L] and [E] are the total concentrations of ligand and enzyme used for the titration, respectively, except for lanosterol, where the better fit was achieved in fitting the data to the Michaelis–Menten equation.

### CYP51 Catalytic Activity Assays

Activity assays were generally performed as previously described for other CYP51 orthologs^31^ using the radiolabeled ([3-^3^H]) sterol substrates lanosterol and obtusifoliol, ∼4000 dpm/nmol. Time-course experiments were carried out at 37 °C at 50 μM concentrations of sterol substrates. For steady-state kinetic analysis, the reactions were run for 60 s for obtusifoliol and 20 min for lanosterol (maximal turnover rates calculated in the time-course experiments), and the sterol concentrations were 3.13, 6.25, 12.5, 25, 37.5, and 50 μM. Michaelis–Menten parameters were calculated using GraphPad Prism. The *k*_cat_ and *K*_m_ values for each reaction were determined by fitting the data to a Michaelis–Menten hyperbola, which was done using GraphPad Prism software, with the reaction rates (nmol product formed/nmol P450/min) being plotted versus total substrate concentration.

### CYP51 Inhibition Assays

Screening for the enzyme inhibitors was carried out using cold obtusifoliol as the substrate, with the previously synthesized CYP51 reaction product of dihydrolanosterol (dihydro-FF-MAS^71^) serving as the internal standard to account for the extraction efficiency for each sample. Briefly, one-hour incubations were performed in 50 mM potassium phosphate buffer, pH 7.4, containing 10% (v/v) glycerol.500 μL samples contained 0.5 μM *A. castellanii* CYP51, 2 μM NADPH-cytochrome P450 reductase, 100 μM L-α-dilauroyl-*sn*-glycero-3-phosphocholine, 50 μM obtusifoliol and 25 μM dihydro-FF-MAS (added from 1 mM stock solutions in 45% (w/v) HPCD), 25 mM sodium isocitrate, 0.4 mg/mL isocitrate dehydrogenase, and various concentration of inhibitors (0, 0.75, 1.5, 3.13, 6.25, 12.5, 25, and 50 μM). The samples were preincubated for 3 min at 37 °C in a shaking water bath, and the reaction was initiated by the addition of 100 μM NADPH and stopped by the extraction of sterols with ethyl acetate. The samples were dried and dissolved in 100 μL of methanol, and the products were analyzed by reversed-phase HPLC using a Nova-Pak 3.9 mm × 150 mm (4 μm) octadecylsilane (C18) HPLC column and Waters 2489 UV/visible detector set at 250 nm. The products were separated at a flow rate of 0.75 mL/min using an isocratic mobile phase composed of acetonitrile and methanol (4:1 (v/v)). The retention times for 14α-desmethyl obtusifoliol and FF-MAS were 11.2 and 17.1 min, respectively (Figure S5). The inhibitory potencies of clinical drugs were first estimated in a 1 h reaction at the inhibitor/enzyme molar ratio 3:1, an approach that we found most helpful in “screening out” less potent compounds.^33,34,74^ Voriconazole and fluconazole were then selected as positive and negative controls. The inhibitory potencies of experimental compounds were compared by determining their “1 h IC_50_” values (concentration required to prevent the conversion of 50% of the substrate in a 1 h reaction). This approach affords the identification of the most potent compounds, which cannot be replaced by the substrate over time, while the initial reaction rates are usually much more strongly affected by most “high-affinity binding ligands”. The values were calculated using GraphPad Prism, with the percentage of substrate converted being plotted against inhibitor concentration and the curves fitted with nonlinear regression (log(inhibitor) vs normalized response).

### Acanthamoeba Cellular Growth Inhibition Assay

*A. castellanii* CDC:0180:1 (ATCC 50491) and *A. polyphaga* (ATCC 30461) were cultured in peptone-yeast extract-glucose (PYG) media at 28 °C. Azole susceptibility assays were performed in 96-well microtiter plates using an adaptation of the resazurin cell respiration assay.^75,76^ Amoeba were grown axenically to confluence in PYG media for 48 h at 28 °C prior to dilution to 5 × 10^4^ trophozoites/mL with PYG media. Serial 100× stock azole antifungal agent dilutions were prepared in DMSO of 6.4, 3.2, 1.6, 0.8, 0.4, 0.2, 0.1, 0.05, 0.025, 0.0125, 0.0625, and 0.03125 mg/mL. These solutions were initially diluted 10-fold using PYG medium, of which 20 μL was added directly to culture plate wells containing 180 μL of trophozoite inoculum (1 × 10^4^ cells) to achieve final azole concentrations of 64, 32, 16, 8, 4, 2, 1, 0.5, 0.25, 0.125, 0.0625, and 0.03125 μg/mL. Control wells containing 1% (v/v) DMSO and trophozoites were prepared. The microtiter plates were incubated at 28 °C for 48 h, after which 30 μL of 0.02% (v/v) aqueous resazurin dye was added, and the microtiter plates were incubated for a further 48 h at 28 °C. A color change from purple to pink indicated the presence of respiring cells. Minimum inhibitory concentration determinations were performed in triplicate.

### SEC Analysis of the Protein Aggregation State

Size-exclusion chromatography was carried out by HPLC using a 30 cm × 10 mm, 8.6 μm particle size Superdex 200 Increase 10/300 GL prepacked gel filtration column (Sigma-Aldrich). The Waters 2489 UV/visible detector was set at 278 and 369 nm. The column was equilibrated with phosphate-buffered saline, pH 7.4 (PBS, Gibco) and calibrated with the protein markers from a Gel Filtration Molecular Weight Markers kit (Sigma) at a flow rate of 0.5 mL/min. The two peaks observed in the *A. castellanii* CYP51 crystallization sample were isolated, and their absorption spectra (278–700 nm) were taken using the Shimadzu UV-2600i spectrophotometer.

### X-ray Crystallography

In an attempt to determine the structure of *A. castellanii* CYP51 in the ligand-free state, a 300 μM P450 sample in 20 mM potassium phosphate buffer, pH 7.4, containing 150 mM NaCl, 10% (v/v) glycerol, 0.1 mM EDTA, and 5.6 mM (v/v) tris(2-carboxyethyl)phosphine (TCEP), was mixed with the detergent *n*-dodecyl-β-d-maltoside (Hampton Research), final concentration of 0.34 mM (2 CMC). The detergent was selected by screening of a Hampton Research Detergent Screen Kit and used to support the protein stability. Prior to crystallization, an absorption spectrum was taken to confirm that the Soret maximum remained at 418 nm, meaning that the P450 remained in the water-ligated form. Crystals were obtained by the vapor diffusion technique in sitting drops overlaid with a reservoir solution consisting of 0.2 M sodium citrate tribasic dihydrate, pH 8.2, and 20% (w/v) PEG 3350. At 25 °C, crystals grew within 2 days.

For cocrystallization of *A. castellanii* CYP51 in complex with VT1161, the 300 μM P450 sample was mixed with a 20 mM solution of VT1161 in DMSO (molar ratio enzyme/inhibitor 1:2), incubated for 30 min at room temperature, and centrifuged at 16,800*g* for 10 min (Prima 1, Midwest Scientific). The solution was transferred to a new tube; *n*-dodecyl-β-d-maltoside was added to 0.34 mM, and an absorbance spectrum was taken to confirm that the Soret maximum was at 422 nm, indicating that the P450 was in the tetrazole-bound state. Crystals were obtained by the vapor diffusion technique in the hanging drops equilibrated against a reservoir solution consisting of 0.1 M sodium citrate, pH 6.7, and 18% (w/v) PEG 8000. At 25 °C, crystals appeared after 17 days.

In both cases, the crystals were cryoprotected with 25% (v/v) glycerol in the corresponding reservoir solution and frozen in liquid nitrogen. The data were collected at 100 K using synchrotron radiation at the Advanced Photon Source, Argonne National Laboratory, the 21-ID-G beamline (wavelength 0.97856 nm). The diffraction images were indexed and integrated with autoProc,^77^ and scaled with Aimless.^78^ The structures were determined by molecular replacement with Phaser-MR in the CCP4 program suite^79^ using 6MIO as the search model for 7UWP. The refinement and model building were performed with Refmac5 (CCP4) and Coot,^80^ respectively. The data collection and refinement statistics are shown in Table S2, and the electron density for the molecules bound in the CYP51 active site is shown in Figure S6. The coordinates and structure factors were deposited in the Protein Data Bank. Structural comparisons were accomplished, and RMSDs were calculated in LSQkab (CCP4) using a secondary structure matching algorithm. The docking of voriconazole was performed in MOE 2022.02.^81^ following the covalent docking of metal-containing enzymes protocol (1,2,4-triazole warhead) with 5 refinement cycles.

## Supporting Information Available

Additional figures illustrating CYP51 sequence alignment; spectral titration curves and response to obtusifoliol; overview of crystal structure 7UWP; sample HPLC profile of *A. castellanii* CYP51 reaction product and FF-MAS; and 2Fo-Fc electron density maps and tables presenting crystallographic data collection and refinement statistics and VT1161-contacting residues in the CYP51 structures across phyla (PDF)
Molecular formula strings for VNI, VFV, LFV, UDO, UDD, VT1161, and VT1598 (CSV)
Voriconazole-bound docking model of 7UWP (PDB)

## Supplementary Material

jm4c00303_si_002.csv

jm4c00303_si_003.pdb

## Abbreviations Used

1 h IC_50_: inhibitor concentration required to prevent conversion of 50% of the substrate in a 1 h reaction
CYP: cytochrome P450
CYP51: sterol 14α-demethylase
dihydro-FF-MAS: (3β,5α)-4,4-dimethyl-cholesta-8,14,24-trien-3-ol
DMSO: dimethyl sulfoxide
GAE: granulomatous amebic encephalitis
HPCD: hydroxypropyl-β-cyclodextrin
LFV: (*R*)-*N*-(1-(2,4-dichlorophenyl)-2-(1*H*-imidazol-1-yl)ethyl)-4-(5-(2-fluoro-4-(2,2,2-trifluoroethoxy)phenyl)-1,3,4-oxadiazol-2-yl)benzamide
MIC: minimal inhibitory concentration
PYG: peptone-yeast extract-glucose
RMSD: root-mean-square deviation
TCEP: tris(2-carboxyethyl)phosphine
VFV: (*R*)-*N*-(1-(3,4′-difluoro-[1,1′-biphenyl]-4-yl)-2-(1*H*-imidazol-1-yl)ethyl)-4-(5-phenyl-1,3,4-oxadiazol-2-yl)benzamide
VNI: (*R*)-*N*-(1-(2,4-dichlorophenyl)-2-(1*H*-imidazol-1-yl)ethyl)-4-(5-phenyl-1,3,4-oxadiazol-2-yl)benzamide
VT1161: (*R*)-2-(2,4-difluorophenyl)-1,1-difluoro-3-(1*H*-tetrazol-1-yl)-1-(5-(4-(2,2,2-trifluoroethoxy)phenyl)pyridin-2-yl)propan-2-ol
VT1598: (*R*)-4-((4-((6-(2-(2,4-difluorophenyl)-1,1-difluoro-2-hydroxy-3-(1*H*-tetrazol-1-yl)propyl)pyridin-3-yl)ethynyl)phenoxy)methyl)benzonitrile
UDD: *N*-(4-(trifluoromethyl)phenyl)-*N*-(1-(5-(trifluoromethyl)pyridin-2-yl)piperidin-4-yl)pyridin-3-amine
UDO: (*S*)-2-(4-chlorophenyl)-2-(pyridin-3-yl)-1-(4-(4-(trifluoromethyl)phenyl)piperazin-1-yl)ethan-1-one
