# Supplementary material for: Identification of Potent and Selective Inhibitors of Acanthamoeba: Structural Insights into Sterol 14α-Demethylase as a Key Drug Target
Source: J Med Chem. 2024 Apr 29;67(9):7443–57. doi: 10.1021/acs.jmedchem.4c00303 (PMC11089504; doi:10.1021/acs.jmedchem.4c00303)
Supplement: Supplementary file 1 — jm4c00303_si_001.pdf [file jm4c00303_si_001.pdf]

Supporting Information

Identification of Potent and Selective Inhibitors of *Acanthamoeba*: Structural Insights into Sterol 14α-Demethylase as a Key Drug Target

Tatiana Y. Hargrove,[†] David C. Lamb,[ŧ] Zdzislaw Wawrzak,[§] Marcus Hull,[ŧ] Steven L. Kelly,[ŧ] F. Peter Guengerich[†], and Galina I. Lepesheva*[,†,ǂ,‡]

[†]*Department of Biochemistry, Vanderbilt University School of Medicine, Nashville, TN, 37232, USA*

[ŧ]*Faculty of Medicine, Health and Life Science, Swansea University, Swansea, SA2 8PP, UK*

[§]*Synchrotron Research Center, Life Science Collaborative Access Team, Northwestern University, Argonne, IL, 60439, USA*

[ǂ]*Vanderbilt Institute of Chemical Biology, Nashville, TN, 37232, USA*

[‡]*Center for Structural Biology, Vanderbilt University, Nashville, Tennessee 37232, USA*

*Corresponding Author: Galina I. Lepesheva, Department of Biochemistry School of Medicine, Vanderbilt University, Nashville, TN, 37232, USA E-mail: galina.i.lepesheva@vanderbilt.edu. Phone: (615) 343-1373

Table of Contents

Multiple sequence alignment of selected CYP51 proteins…………………………………………………………. Figure S1

Spectral titration curves for the binding of various heterocyclic ligands to *A. castellanii* CYP51……………………… Figure S2

Obtusifoliol-induced spectral response in truncated *A. castellanii* CYP51……………………………………………….Figure S3

Overview of the crystal structure of detergent-bound *A. castellanii* CYP51 (7UWP)……………………………………Figure S4

HPLC profile of *A. castellanii* CYP51 reaction product and FF-MAS used as the internal standard…………………… Figure S5

The 2Fo-Fc electron density map for the molecules bound in the CYP51 active site……………………………… Figure S6

Crystallographic data collection and refinement statistics …………………………………………………………………..Table S1

VT1161-contacting residues in the CYP51 structures across phyla……………………………………………………...Table S2

Figure S1. Multiple sequence alignment of selected CYP51 proteins from different biological kingdoms. The alignment was performed using Clustal Omega (https://www.ebi.ac.uk/Tools/msa/clustalo/)

```
                         *        20         *        40         *        60         *        80         *       100
A.castellanii  :~~~~~~~~MVA---AAAL--------EL----------FSAHPFIFGPLVLVALLA-LIVKVREWRKYGGYNLPPVVSSLI--PFVGSGLSFAGGPLQYTTDA :71
A.polyphaga    :~~~~~~~~MVA---AAAL--------EL----------FSAHPFIFGPLVLVALLA-LIVKVREWRKYGGYNLPPVVSSLI--PFVGSGLSFAGGPLQYTTDA :71
G.partita      :~~~~~~~~MLS-HDSAALSTFMSS-LEA---YCWTLVYFLSTILFLGIFWRISGSF-FLSKLGLSREVKGKQLPPTFQEGL--PLIGNLIAFAKGPLTVVQKG :87
G.sulphuraria  :~~~~~~~~MLS-QDSIALSTLTSS-LEA---YCWALVYILSTILFFGILWRITGSF-FLSKLGIAREVKGQQLPPTYKEGL--PLVGNLIAFAKGPLNVVQRG :87
G.theta        :~~~~~~~~~~~~~~~~~~~~~~~~~~~~~~~~~~~~~~~~~~~~~~~~MDRVGGVWLLWKRLKQYLEYRGRKLPPVYREGLHVPLLGAALQFLSDPLKMARRA :55
T.trahens      :~~~~~~~~~~~~~~~~~~~~~~~~~~~~~~~MLNTLLTLAS-----NPYVVIGVVA-AILLFVLFIGFTPANAPPIVSTGL--PIVGPILAFMKNPYQLALDS :64
C.sorokiniana  :~~~~~~~~~MA-DHLQAV---VAA-LQA---------LPLAAQVAVAVGSTLVL---AVCLRLLKHTVLR-GSAPPVEEGI--PFIGGLLKFSKGPLPLMEEM :74
P.miyajii      :~~~~~~~~~~~~~MVAAA---VAS-LAA---------LPASTLIG---GAVLLLVV-PAVFVRATANLRR-GNGPPVDEGI--PFVGGLLAFSKGPWYLMQTM :70
O.lucimarinus  :~~~~~~~~~~~~~~~~~~~~~~~~~~~~~~~~~~~~~~~~~~~~~MT--VVIF--I-FLLDQ-LSHGPLKGRKSPPVIDVA--PVWGGMLAFLAGPMKLMREA :50
V.reticuliferus:~~~~~~~~~MA--ELTAE---LSV-LLE---------KINIPQLMLGASAVLL--L-ALVIGRLLFNNFP-GKRPPVYEGI--PYVGGLIKFAKGPWKLLHDG :73
G.pectorale    :~~~~~~~~~MA--DLAAE---LSA-LVA---------NYSTAQLALASGAVLF--L-ALMVGRLLFNNLP-GKRPPVFEGI--PYIGGLIKFAKGPWNLLEEG :73
G.rivale       :~~~~~~~~MQI----SNP---MMG------------SDGGAIPIVA--TVPL--A-VLIAVILIRRRSGYRLPP-TVSAF--PVVGGLMRFLNGPIVMIREE :67
P.californicum :~~~~~~~~MDF----TNL---PMD-LKE-------IKLFNVGILIVA--TLIV--A-KILSSLFMSGSKK-NLPP-VVKSW--PLLGGLARFMKGPIIMLREE :71
V.vinifera     :~~~~~~~~~~~~~~~~~~~~~~MD--VD-------NKFFNVALLIVA--TVVV--A-KLISALLIPKSRK-RLPP-TVKAF--PVIGGLLRFLKGPVVMLREE :63
L.tenue        :~~~~~~~~MDW----DPE---N-N-KLS-------PKLVNVGLLIVA--TVLL--A-KVVSALFMSRSGKRRLPPRVKQGI--PVVGGLLRFLKGPIVMLREE :72
N.fowleri      :~~~~~~~~~~~~~~~~~~~~~MSS-E------------IGVLDIVIALSTALV--V-FLVIRYFSSQKGKKNEPPRVPNLI--PYVGSFVSFAKNPVQFIIDN :64
Sea urchin     :~~~~~~~MANSVNIFESVGGVFGE-------MTLATMILVSTIFVLGIAW--------AFKSLLGPQNKDVKLPPRLPTGI--PFLGQAVAFNQSPIDFLEDA :79
Salmon         :~~~~~~~~~MAMHLYQVSSMLIENTV-GKMSDNLTSVVLAVSVITLTLGYISK----LVLKQSQSSSEEHKKYPPYIPSSI--PFLGHAIAFGKSPIEFLENA :87
Toad           :~~~~~~~~~MLVNLWQAGGAL----LEEAVGDSLTSKVLLPWAFLLTIAYLSK----LAFKHLHTIDTDNLKYPPYISSSI--PFLGHAISFGKSPIEFLERA :84
Barn owl       :~~~~~~~~~~MLSLMEVGG----SLLERVAVGNPLSLVLAASAFALSLGYLFQ----LGYRRHVGS--DQRKYPPYISSSI--PFLGHAIAFGKSPIEFLENA :81
Human          :~~~~~~~~MLLLGLLQAGGSVLGQAMEKVTGGNLLSMLLIACAFTLSLVYLIR----LAAGHLVQL-PAGVKSPPYIFSPI--PFLGHAIAFGKSPIEFLENA :88
T.brucei       :~~~~~~~~~~~~~~~~~~~~~~~~~~~~~~~~~~~~~~~~~~MLLEVAIFLLTALALYSFYFVKSFNVTRPTDPPVYPVTV--PILGHIIQFGKSPLGFMQEC :59
T.cruzi str.Y B:~~~~~~~~~~~~~~~~~~~~~~~~~~~~~~~~~~~~~~~~~~MFIEAIVLALTALILYSVYSVKSFNTTRPTDPPVYPVTV--PFLGHIVQFGKNPLEFMQRC :59
T.cruzi str.Y A:~~~~~~~~~~~~~~~~~~~~~~~~~~~~~~~~~~~~~~~~~~MFIEAIVLGLTALILYSVYSVKSFNTTRPTDPPVYPVTV--PFLGHIVQFGKNPLEFMQRC :59
C.neoformans   :MS-------AI-IPQVQ-QLLGQVAQFF--PPWFAALPTSLKVAIAVVGIPALIIGLNVFQQLCLP-RRKDLPPVVFHYI--PWFGSAAYYGEDPYKFLFEC :88
S.cerevisiae   :MSATKSIVGEAL-EYVNI-G-----------LSHFLA----LPLAQRISLIIIIPFIYNIVWQLLYSL-RKDRPPLVFYWI--PWVGSAVVYGMKPYEFFEEC :83
A.fumigatus B  :~~~~~~~~MGLI-AFILD-G-ICKHCSTQ--S---TW----VLVGIGLLSILAVSVIINVLQQLLFK--NPHEPPVVFHWF--PFIGSTISYGIDPYKFFFDC :79
N.fischeri B   :~~~~~~~~MGLI-AFILD-G-ICKHCSTQ--S---TW----VLVGVGLLSILAVSVIANVLQQLLFK--NPHEPPVVFHWF--PFIGSTISYGIDPYKFFFDC :79
F.graminearus B:~~~~~~~~MGLL-QELAG-HPLAQQFQEL--P---LG----QQVGIGFAAFLVLSVVLNVLNQLLFR--NPNEPPMVFHWF--PFVGSTITYGMDPPTFFREN :80
O.sinensis B   :~~~~~~~~MGLV-SAVGS-HPLAQQFQAL--G---FA----WQTAIALGGFLALSVMLNVANQVLNK--NPLEPPLVFHWF--PFVGSTITYGMDPPRFFQEN :80
A.fumigatus A  :~~~~~~~~~~~~~~~~~~~~~~~~~~~~~~~~~MVPM----LWLTAYMAVAVLTAILLNVVYQLFFRLWNRTEPPMVFHWV--PFLGSTISYGIDPYKFFFAC :64
N.fischeri A   :~~~~~~~~~~~~~~~~~~~~~~~~~~~~~~~~~MVPM----LLLTAYMAVAMLTAILLNVVYQLFFRLWNRTEPPMVFHWV--PFLGSTISYGIDPYKFFFAC :64
F.graminearus A:~~~~~~~~~~~~~~~~~~~~~~~~~~~~~~~~~MFHL----LIYPLWVLVALFAVIIANLLYQQLPR--RPDEPPLVFHWF--PFFGNAVAYGLDPCGFFEKC :62
O.sinensis A   :~~~~~~~~~~~~~~~MSS-SLFHELFLRM--GP-----------GSLVFGLAFCFVIGNILWQLLPK--SRSEPPLVFHLI--PFVGNAVSYGMDPFKFYTEC :70
D.loculata A   :~~~~~~~~~~~~~~~~~~~~~~~~~~~~M--INLNTP----THNVAYVIATLILTIAINVLWQLLPR--RKSEPPLVFHWI--PFVGSAISYGLNPFEFFTNC :65
M.brunneum A   :~~~~~~~~~~~~~~~MEF-RPFTEHLKRM--AYVSPI----LFLGSCLLLLVLLLIVVNLIRQQLPA--SKSEPPLVFHWI--PFIGNAVSYGLDPFAFYSQC :77
                                                                                         pP        P  G    5   P
```

```
                       *       120         *       140         *       160         *       180         *       200
A.castellanii  :YKK-YGDIFTMKVFGQRLTFLVGPDAHVPFFSQGDAELSQDEPYQ-FSVPIFGPNVVYGADLAHR-NQQL--KFIAASLSTKALQSYVPLIVKEAEDFFAKW- :168
A.polyphaga    :YKK-YGDIFTMKVFGQRLTFLVGPDAHVPFFSQGDAELSQDEPYQ-FSVPIFGPNVVYGADLAHR-NQQL--KFIAASLSTKALQSYVPLIVKEAEDFFAKW- :168
G.partita      :YQS-CGDIFTFKVLHKHITFLVGPKAHEVFFQGTDDELDQNEVYA-FSVPIFGKGVVYDAPLDKR-LQQL--RIMSAALRPTRMYGYVDQMVHEAVQFFRKW- :184
G.sulphuraria  :YQS-CGDIFTFKVFHKHITFLVGPKAHEIFFQGTDDELDQNEVYA-FSVPIFGKGVVYDAPLEKR-LQQL--RIMSAALRPARMYGYVDQMVLEAVQFFRKW- :184
G.theta        :QDV-HGDIFTVYVLGKRLTFMCGPDAQENFCRARDDELSQQQAYK-FSVPLFGPGVVYDADLEKR-TEQI--KFVTNSLHTTAIGQYVPMMVDEAEKYFASW- :152
T.trahens      :YRR-YGEVFTIKVANQRLTFLVGPEASEPFFKGKDADMSQNEVYK-FSVSIFGKGVVYDVPTPIR-IEQI--KFMSSALRSDVLRSYVDDMVLEARQYFGKW- :161
C.sorokiniana  :YKK-HGEVFTIPLVHKNMTFVIGPHASPHFFNATDDKMSQTEVYN-FNVPTFGKGVVYDVDQRVR-SEQF--RFVADALRTAKLRTYVPAFKQEAEEYFSKW- :171
P.miyajii      :YKK-HGEVFTVPLAHRRMTFLIGPHASPHFFNATDDKMSQTEVYN-FNVPTFGPGVVFDVDQRVR-SEQF--RFVADALKSSKLKTYVPAFHKEAEEYFSQW- :167
O.lucimarinus  :TPK-YGEVFTVPVFHKRITFLIGPKVSEFFFKAKDTEMSQKEVYE-FNVPTFGKGVVFDVDHTTR-AEQF--RFFADSLKSNRLRMYVGMMVKEAEDFFSKW- :147
V.reticuliferus:YAN-FGEVFTVPVAHKRVTFLIGPDVSPHFFKAGDDEMSQSEVYD-FNVPTFGPGVVFDVEQKIR-TEQF--RMFTEALTKNRLKAYVPQFNKEAEEYFAKW- :170
G.pectorale    :YAK-HGEVFTVPVAHKRVTFLIGPEVAPHFFKAGDDEMSQSEVYD-FNVPTFGKGVVFDVDQKIR-TEQF--RMFTEALTKNRLKSYVPHFNKEAEEYFAKW- :170
G.rivale       :YPK-LGSVFTLNLFHKRITFLIGPEVSAHFFKAPESELSQQEVYQ-FNVPTFGPGVVFDVDYTVR-QEQF--RFFTEALRVNKPKGYVDQMVTEAEDYFSKW- :164
P.californicum :YPK-LGSVFTVNLVNKKITFFIGPEVSSHFFKAPETDLSQQEVYQ-FNVPTFGPGVVFDVDYSVR-QEQF--RFFTESLRVTKLKGYVEQMVSEAEDYFSKW- :168
V.vinifera     :YPK-LGSVFTLNLLNKNITFFIGPEVSAHFFKAPEADLSQQEVYQ-FNVPTFGPGVVFDVDYSVR-QEQF--RFFTESLRVTKLKGYVDQMVTETEDYFSKW- :160
L.tenue        :YPK-LGSVFTLNLVNRNITFLIGPEVSAHFFKAPEADLSQQEVYQ-FNVPTFGPGVVFDVDYSIR-QEQF--RFFTESLRVNKLKGYVDMMVTEAQDYFSKW- :169
N.fowleri      :SKK-YGDVFTATILGKEMTFLNHPKILDTFFKATDNELSLRDVYR-FMRPVFGTGVVYDADSTERMMEQV--KFVSSGLTTARFRVFVDIFEDEIAHKVKEL- :162
Sea urchin     :YEK-YGDVFSFTMVGKTFTYLIGSEASALLFNSKNENLNAEEVYSNLTVPVFGKGVAYDVPNPVF-VEQK--KMLKTGLNIQQFKRHIPLIEEETREYFNRW- :177
Salmon         :YEK-YGPVVSFTMVGKTFTYLLGSEAATLMFNSKNEDLNAEDVYSRLTTPVFGKGVAYDVPNHIF-LEQK--KMFKTGLNIAHFKQHVEIIEEETKEYFSRW- :185
Toad           :YEK-YGPVFSFTMVGKTFTYLVGSDAAALLFNSKNDDLNAEDVYSRLTTPVFGKGVAYDVPNPIF-LEQK--KMFKTGLNIAHFKNHVRMIEEETVDYLKRW- :182
Barn owl       :YDK-YGPVFSFTMVGKTFTYLLGSDAAALLFNSKNEDLNAEDVYSRLTTPVFGKGVAYDVPNTVF-LEQK--KMLKTGLNIAQFKQHVSMIEEETKEYFKAW- :179
Human          :YEK-YGPVFSFTMVGKTFTYLLGSDAAALLFNSKNEDLNAEDVYSRLTTPVFGKGVAYDVPNPVF-LEQK--KMLKSGLNIAHFKQHVSIIEKETKEYFESW- :186
T.brucei       :KRQLKSGIFTINIVGKRVTIVGDPHEHSRFFLPRNEVLSPREVYS-FMVPVFGEGVAYAAPYPRM-REQL--NFLAEELTIAKFQNFVPAIQHEVRKFMAAN- :157
T.cruzi str.Y B:KRELKSGVFTISIGGQRVTIVGDPHEHSRFFSPRNEILSPREVYT-IMTPVFGEGVAYSAPYPRM-REQL--NFLAEELTIAKFQNFVPAIQHEVRKFMAEN- :157
T.cruzi str.Y A:KRDLKSGVFTISIGGQRVTIVGDPHEHSRFFSPRNEILSPREVYT-IMTPVFGEGVAYAAPYPRM-REQL--NFLAEELTIAKFQNFVPAIQHEVRKFMAEN- :157
C.neoformans   :RDK-YGDLFTFILMGRRITVALGPKGNNLSLGGKISQVSAEEAYTHLTTPVFGKGVVYDCPNEML-MQQK--KFIKSGLTTESLQSYPPMITSECEDFFTKEV :187
S.cerevisiae   :QKK-YGDIFSFVLLGRVMTVYLGPKGHEFVFNAKLADVSAEAAYAHLTTPVFGKGVIYDCPNSRL-MEQK--KFVKGALTKEAFKSYVPLIAEEVYKYFRDSK :182
A.fumigatus B  :RAK-YGDIFTFILLGKKTTVYLGTKGNDFILNGKLRDVCAEEVYSPLTTPVFGRHVVYDCPNAKL-MEQK--KFVKYGLTSDALRSYVPLITDEVESFVKNSP :178
N.fischeri B   :RAK-YGDIFTFILLGKKTTVYLGTKGNDFILNGKLRDVCAEEVYSPLTTPVFGRHVVYDCPNAKL-MEQK--KFVKYGLTSDALRSYVPLITDEVESFVKNSP :178
F.graminearus B:RAK-HGDVFTFILLGKKTTVAVGPAGNDFILNGKLKDVCAEEIYTVLTTPVFGKDVVYDCPNAKL-MEQK--KFMKIALTTEAFRSYVPIISSEVRDYFKRSP :179
O.sinensis B   :RAK-HGHVFTFVLLGKKTTVAVGPAGNDFILNGRLKDVNAEEIYSVLTTPVFGRDVVYDCPNAKL-MEQKKARFMKIALTTEAFRSYVPIISDEVQSYFKRDA :181
A.fumigatus A  :REK-YGDIFTFILLGQKTTVYLGVQGNEFILNGKLKDVNAEEVYSPLTTPVFGSDVVYDCPNSKL-MEQK--KFIKYGLTQSALESHVPLIEKEVLDYLRDSP :163
N.fischeri  A  :REK-YGDIFTFILLGQKTTVYLGVQGNEFILNGKLKDVNAEEVYSPLTTPVFGSDVVYDCPNSKL-MEQK--KFIKYGLTQSALESHVPLIEKEVLDYLRNSP :163
F.graminearus A:REK-HGDVFTFILFGRKIVACLGVDGNDFVLNSRLQDANAEEVYGPLTIPVFGSDVVYDCPNSKL-MEQK--KFVKFGLTQKALESHVQLIEREVLDYVETDP :161
O.sinensis A   :RQK-HGDIFTFVLFGRKMTVYLGVDGNDFILNGKLQDLNAEEIYAPLTTPVFGKDIIYDCPNAKL-MEQK--RFVKFGLTRAALCSYVGMIEQETIDYLESAP :169
D.loculata A   :RKH-HGDIFTFVLFGKKITVYLGVEGNEFILNGKLRDVNAEDIYGALTTPVFGKDIIYDCPNEKL-MEQK--KFVKFGLTQKALESYVPLIEQEVLEYIKAYP :164
M.brunneum A   :QKK-HGNIFTFVLFGRKMTVYLGLQGNDFILNGKLQDVNAEEIYAPLTTPVFGSDIIYDCPNAKL-MEQK--KFVKVGLTHDALCSYVPLIEKEVVDYLKVAP :176
                     g 6f3  6     t   g                    Y     p FG  6 5d        2Q         L              E
```

```
                    *        220         *        240         *        260         *        280         *        300
A.castellanii  :------DKSGTVDIRDALAELIILTASRCLMGKEIRENLFT-EVAKLYQTLDEGLLPISVFFP-Y---LPIPAHKRRDEARLAMVRMFKKIIDERRANPEV-- :258
A.polyphaga    :------DKSGTVDIRDALAELIILTASRCLMGKEIRENLFT-EVAKLYQTLDEGLLPISVFFP-Y---LPIPAHKRRDEARLAMVRMFKKIIDERRANPEV-- :258
G.partita      :------GEQGEVDILESLSDLIILTASRCLMGREVREQLFE-KVSKLYHDLDQGMQPISVFAP-Y---LPISAHRKRDKAREEMVQLFRTVIQNRRMR-GL-- :273
G.sulphuraria  :------GDQGQVDILESLSDLIILTASRCLMGREVREQLFE-KVSKLYHDLDQGMQPISVFAP-Y---LPISAHRKRDKAREEMVQLFRTVIQNRRRR-NV-- :273
G.theta        :------GDEGVVDIYTALAELIILTASRCLMGPEIRSELHK-EVSELYAILDKGCTPISFFAP-Y---FPIPAHFRRDKARRQMVALFQKVIEKRRKE-NK-- :241
T.trahens      :------GESGTVDLAQVFSELIILTASRCLMGPEVREHVFE-QVAEIFRALDEGLLPITFFFP-E---LPIPAHRARNAAREEMVKLFAGIMEERRANPDK-- :251
C.sorokiniana  :------GDTGVVDLMQTFSDLIILTASRTLLGREVRENMFK-EVADLYHDLDDGMRPISVLFP-Y---LPTEYHRKRDAAREALRAIFAKIITARRAN-NV-- :260
P.miyajii      :------GETGVVNIAQEFGQLIILTASRTLLGREVRESMFQ-RVADLFHDLDDGMRPLSVLFP-Y---LPTAYHKKRDAARKELSQIFRKIVAARRAS-GV-- :256
O.lucimarinus  :------GDAGEVDLLEQLSELIVLTASRCLLGREIRETLYS-EVTDLVHDLDKGMVPISVFFP-Y---APIEAHRKRDAARKNLAKIFDKVIQARRES-GA-- :236
V.reticuliferus:------GDEGIIDFRDEFSKLVTLTAARTLLGREVREQLFE-QVADLLHGLDEGMVPISVFFP-Y---LPIPVHLKRDRCRKELSTIFGKVIRHRRES-GH-- :259
G.pectorale    :------GDTGVIDFKDEFCKLVTLTAARTLLGREVREHLFE-QVADILHGLDEGMVPISVFFP-Y---APIPAHWKRDRCRKQLSEIFAKVIRQRRAS-NT-- :259
G.rivale       :------GESGEVDLKYELEHLIILTASRCLLGREVRDKLFD-DVSALFHDLDNGMLPISVIAP-Y---LPIAAHRRRDQARKKLSSIFANIINSRKRA-AE-- :253
P.californicum :------GESGEVDLKYELEHLIILTASRCLLGQEVRDQLFD-DVSALFHDLDAGMLPISVIFP-Y---LPIPAHRRRDQARKKLARIFARIIASRKQT-GK-- :257
V.vinifera     :------GDSGEVDLKYELEHLIILTASRCLLGQEVRDKLFA-DVSALFHDLDNGMLPISVIFP-Y---LPIPAHRRRDQARTKLAHIFANIIASRRET-GK-- :249
L.tenue        :------GDSGEVDLKYELEHLIILTASRCLLGREVRDKLFD-DVSALFHDLDNGMLPISVLFP-Y---LPIPAHRRRDLARKKLAQIFASIINSRKKT-GS-- :258
N.fowleri      :------GPEGTVDVAELMADLIIFTASRCLLGDEVRQYLSEKNLGKLYHDIDDGISPLSFFYP-S---LPAP---KRDKARKAVGEIFQELLDKRREEHKKHP :252
Sea urchin     :------GDSGEKNLFVALSELIILTASRCLHGKEIRSMLDE-EVAQLYADLDGGFTHLAWLAPSW---IPFPSFLRRDRAHRKIKQIFYKAIAKRRET-G--- :266
Salmon         :------GDSGEQNLFEALSELIILTASACLHGKEIRSMLDE-KVAQLYADLDGGFSHAAWLLPGW---LPLPSFRRRDRAHREIKNIFYKVITQKRSS-GE-- :275
Toad           :------GDSGVGNLFEALSELIILTASLCLHGREIRSLLDE-KVAQLYADLDGGFTHAAWLLPGW---LPLPSFRRRDRAHREIKNIFYQVIQKRRQS-QD-- :272
Barn owl       :------GESGEKNLFEALSELIILTASHCLHGKEIRSLLNE-KVAQLYADLDGGFTFAAWLLPGW---LPLPSFRRRDRAHKEIKNIFYKVIQKRRKS-EE-- :269
Human          :------GESGEKNVFEALSELIILTASHCLHGKEIRSQLNE-KVAQLYADLDGGFSHAAWLLPGW---LPLPSFRRRDRAHREIKDIFYKAIQKRRQS-QE-- :276
T.brucei       :-WD---KDEGEINLLEDCSTMIINTACQCLFGEDLRKRLDARRFAQLLAKMESSLIFAAVFLP-ILLKLPLPQSARCHEARTELQKILSEIIIARKEE----- :250
T.cruzi str.Y B:-WK---KDEGVINLLEDCGAMIINTACQCLFGEDLRKRLNARHFAQLLSKMESSLIPAAVFMP-WLLRLPLPQSARCREARAELQKILGEIIVAREKE----- :250
T.cruzi str.Y A:-WK---EDEGVINLLEDCGAMIINTACQCLFGEDLRKRLNARHFAQLLSKMESSLIPAAVFMP-WLLRLPLPQSARCREARAELQKILGEIIVAREKE----- :250
C.neoformans   :GISPQ-KPSATLDLLKAMSELIILTASRTLQGKEVRESLNG-QFAKYYEDLDGGFTPLNFMFP-N---LPLPSYKRRDEAQKAMSDFYLKIMENRRKG-ES-- :281
S.cerevisiae   :NFRLNERTTGTIDVMVTQPEMTIFTASRSLLGKEMRAKLDT-DFAYLYSDLDKGFTPINFVFP-N---LPLEHYRKRDHAQKAISGTYMSLIKERRKN-NDI- :278
A.fumigatus B  :AFQ---GHKGVFDVCKTIAEITIYTASRSLQGKEVRSKFDS-TFAELYHNLDMGFAPINFMLP-W---APLPHNRKRDAAQRKLTETYMEIIKARRQA-GSK- :271
N.fischeri B   :AFQ---GHKGVFDVCKTIAEITIYTASRSLQGKEVRSKFDS-TFAELYHNLDMGFAPINFMLP-W---APLPHNRKRDAAQRKLTETYMEIIKARRQT-GSK- :271
F.graminearus B:DFK---GKSGIADIPKKMAEITIFTASHALQGSAIRSKFDE-SLAALYHDLDMGFTPINFMLH-W---APLPWNRKRDHAQRTVAKIYMDTIKERRAK-GNN- :272
O.sinensis B   :DFK---AKSGIVDIPKKMAEITIFTASHALQGSAIRNKFDT-SLAALYHDLDMGFTPINFMLH-W---APLPWNRKRDHAQRTVAKIYMDTINERRAQ-GAA- :274
A.fumigatus A  :NFQ---GSSGRMDISAAMAEITIFTAARALQGQEVRSKLTA-EFADLYHDLDKGFTPINFMLP-W---APLPHNKKRDAAHARMRSIYVDIINQRRLD-GDK- :256
N.fischeri A   :NFQ---GSSGQVDISAAMAEITIFTAARALQGQEVRSKLTA-EFADLYHDLDKGFTPINFMLP-W---APLPHNKKRDAAHARMRSIYIDIINQRRLD-GEK- :256
F.graminearus A:SFS---GRTSTIDVPKAMAEITIFTASRSLQGEEVRRKLTA-EFAALYHDLDLGFRPVNFLFP-W---LPLPHNRKRDAAHIKMREVYMDIITDRRKG-GIR- :254
O.sinensis A   :AFR---GRCGSVNIPAAMAEITIYTAGRALQGVEVRSKLTA-EFADLYHDLDLGFRPINFLVP-W---APLPQNRRRDVAHTKMEAIYLDIINKRRQS-GKP- :262
D.loculata A   :SFK---GSSGIVDIPAAMAEITIFTASRALQGAEVRKKLTA-EFADLYHDLDLGFQPINFLMP-W---APLPQNRRRDVAHAKMTETYLELINNRRAD-KKSK :258
M.brunneum A   :AFR---GQSGTVNIPAAMAEITIFTASRTLQGREVRGKLSA-EFAELYHDLDLGFRPINFLMP-W---APLPQNRRRDAAHAKMRSIYIDIINDRRVS-GKV- :269
                         g 1     6  TA  L G e6R       l  6d g p    p    P p   r a 6        R

                    *        320         *        340         *        360         *        380         *        400         *
A.castellanii  :------KHNDCLQVFM-DARYR---GEEQALNDEEITGLMIALLFAGQHTSSVTGSWTGLLLFEANN--------KKKFLPGVLEEQEEIRKEFG------DE :337
A.polyphaga    :------KHNDCLQVFM-DARYR---GEEQALNDEEITGLMIALLFAGQHTSSVTGSWTGLLLFEANN--------KKKFLPGVLEEQEEIRKEFG------DE :337
G.partita      :------KEDDMLQTFM-DASYR---DGSRP-SEFEVAGLLIALLFAGQHTSSITGSWTGMLMLRNK-----------SIFERVKKEQDAIIEEFG------DE :348
G.sulphuraria  :------KEDDMLQTFM-DASYR---DGSRP-SEYEVAGLLIALLFAGQHTSSITGSWTGMLMLRNK-----------DVFERVKKEQDTIIEEHG------DE :348
G.theta        :------RYNDVLQVYM-DATYK---NGDK-LPAHEVTGLLIALLFAGQHTSSVTSSWTGLYMLDNKK----------DIIPKLVEEQK----KVG------KV :313
T.trahens      :------IYTDMLDQFM-NAQYK---DGRT-LTDREVTGLMISLLFAGQHTSSNSSASTLLHLLRSDSTVD-----NTEHLQAVLDEQQAMIAEFG------DE :332
C.sorokiniana  :------KEDDILQVLI-DSRYKNVYGGRA-TNDSEITGLLIAILFAGQHTSSVTSSWTGYRMLS-NR----------KWFEAAQEEQRRVMREHG------DK :338
P.miyajii      :------KEDDVLQALI-DSRYKVYDGRA-TTDEEITGLLIALLFAGQHTSSMVSTWTGLYLASGDQ-----------RYYRAAEEEQRRVMAKHG------DR :335
O.lucimarinus  :------SEPDVLQTFI-DARYK---DGSR-LTNDQVLGMLIAVLFAGQHTSSITSTWTGLLAIANKE----------RVMPALEKEQKDIMKKHG------KD :312
V.reticuliferus:------REEDVLQQFI-DARYQNVNGGRA-LTEEEITGLLIAVLFAGQHTSSITTSWTGIFMAANKE----------KAWLPAVEEQKAIVAKHG------TD :338
G.pectorale    :------REEDVLQQFC-DAKYQNVLGGRA-LTEEEITGLLIAVLFAGQHTSSITTSWTGIFMAANKD----------KAWNPAVEEQKAIIAKHG------TT :338
G.rivale       :------SENDMLQCFI-DSKYK---NGRS-TTESEVTGLLIAALFAGQHTSSITSTWTGAYLLQNK-----------KYLAAVPEEQQRLMEKHG------KK :328
P.californicum :------CENDMLQCFI-DSKYK---DGRP-TTELEVTGLLIAALFAGQHTSSITSVWTGAYLLFKNK----------QFLSGVVDEQKNLMKKHG------GK :332
V.vinifera     :------SENDMLQCFM-DSKYK---DGRQ-TTEAEVTGLLIAALFAGQHTSSITSTWTGAYLLFRHK----------EFLSAVLDEQKNLMKKHG------NK :324
L.tenue        :------SENDMLQCFI-DSKYK---DGRP-TTEGEVTGLLIAALFAGQHTSSITSTWTGAYLLRHKE----------EYLSSVVEEQKQLLRKHG------NR :334
N.fowleri      :ERLLDESKMDVVDHLI-TQKYK---DGQE-LTDVHRIGILIAGLFAGQHTSSITSSWTLMNVISNK-----------KVLEKVRKEQEEIMG-SD------KV :332
Sea urchin     :------VENDMLQTLI-DSKYK---SGRP-LSDDEIAGMCIGLLLAGQHTSSTTSSWTGFFLARDK-----------EVQDRVLEEQIKVCGDAS------TE :341
Salmon         :------KVDDMLQTLI-DATYK---DGRP-LNDDEIAGMLIGLLLAGQHTSSTTSSWLGFFLGKDK-----------ALQDRCYAEQKTACGEDL------PP :350
Toad           :------QEDDMLQTLI-DATYK---DGTP-LTDDEIAGMLIGLLLAGQHTSSTTSAWMGFFLAKNK-----------SIQDQCFAEQKAVCGEDL------PP :347
Barn owl       :------KEDDMLQTLI-DASYK---DGRP-LTDDEIAGMLIGLLLAGQHTSSTTSAWLGFFLARDK-----------SIQEQCYAEQKAVCGDDL------PP :344
Human          :------KIDDILQTLI-DATYK---DGRP-LTDDEVAGMLIGLLLAGQHTSSTTSAWMGFFLARDK-----------TLQKKCYLEQKTVCGENL------PP :351
T.brucei       :EVNKDSSTSDLLSGLL-SAVYR---DGTP-MSLHEVCGMIVAAMFAGQHTSSITTTWSMLHLMHPA----------NVKHLEALRKEIEE----------FPAQ :329
T.cruzi str.Y B:EASKDNNTSDLLGGLL-KAVYR---DGTR-MSLHEVCGMIVAAMFAGQHTSTITTSWSMLHLMHPK---------NKKWLDKLHKEIDE----------FPAQ :329
T.cruzi str.Y A:EASKDNNTSDLLGGLL-KAVYR---DGTR-MSLHEVCGMIVAAMFAGQHTSTITTSWSMLHLMHPK---------NKKWLDKLHKEIDE----------FPAQ :329
C.neoformans   :-----DHEHDMIENLQ-SCKYR---NGVP-LSDRDIAHIMIALLMAGQHTSSATSSWTLLHLADRP-----------DVVEALYQEQKQKLGNPDGT--F-RD :360
S.cerevisiae   :------DDELDLSLMKNSTYK---DGVK-MTDQEIANLLIGVLMGGQHTSAATSAWILLHLAERP-----------DVQQELYEEQMRVLD---GG-K-KE :354
A.fumigatus B  :-----KDSEDMVWNLM-SCVYK---NGTP-VPDEEIAHMMIALLMAGQHSSSSTASWIVLRLATRP-----------DIMEELYQEQIRVLGSD-----LP-P :347
N.fischeri B   :-----KDSEDMVWNLM-SCVYK---NGTP-VPDEEIAHMMIALLMAGQHSSSSTASWIVLRLATRP-----------DIMEELYQEQIRVLGSD-----LP-P :347
F.graminearus B:-----ESEHDMMKHLM-NSTYK---NGIR-VPDHEVAHMMIALLMAGQHSSSSTSSWIMLRLAQYP-----------HIMEELYQEQVKNLGAD-----LP-P :348
O.sinensis B   :-----DDELDIMKHLM-SSKYK---NGTP-VPDHEVAHMMIALLMAGQHSSSSTSSWIMLRLAQYP-----------DMMEELYQEQVKVLGAH-----LP-P :350
A.fumigatus A  :----DSQKSDMIWNLM-NCTYK---NGQQ-VPDKEIAHMMITLLMAGQHSSSSISAWIMLRLASQP-----------KVLEELYQEQLANLGPAGPDGSLP-P :338
N.fischeri A   :----DSQKSDMIWNLM-NSTYK---NGQQ-VPDKEIAHMMITLLMAGQHSSSSISAWIMLRLASQP-----------KVLEELYQEQLANLGPVGPDGSLP-P :338
F.graminearus A:----TEDGTDMIANLM-GCTYK---NGQP-VPDKEIAHMMITLLMAGQHSSSASSWIVLHLASSP-----------DITEELYQEQLVNLSVN---GALP-P :333
O.sinensis A   :---PDDDDVDMIWHLM-TCVYK---NGTP-VPDMEIAHMMITLLMAGQHSSSSSSWIMLRLASRP-----------DIVEELYHEQVRILGYD---AALPRP :343
D.loculata A   :EASADTDGTDMILNLM-GSIYK---DGQV-LSDENIAHMMITLLMAGQHSSSSSAWIMLRLASRP-----------DIAEELYHEQVNNIHYT---G---QP :339
M.brunneum A   :---PDQ-DADMIWHLM-TCVYK---DGTP-LPDKEIAHMMITLLMAGQHSSSSSSWIMLRLASRP-----------DIVEELYQEQVRNLGYT---GS--EP :347
                             D 6        Y    g          6 6   6 aGQH3S     w                          Eq
```

```
                        420         *        440         *        460         *        480         *        500         *
A.castellanii   :LTMEA-LNKMDKLHRCVKEALRMYPPLLFVMRKVIKPFSY----------KDYYVPEGDTVFVSPALSMREEVFPNA-DQYNPERFVEED--K--------- :417
A.polyphaga     :LTMEA-LNKMDKLHRCVKEALRMYPPLLFVMRKVIKPFSY----------KDYYVPEGDTVFVSPALSMREEVFPNA-DQYNPERFVEED--K--------- :417
G.partita       :LNYDV-LSKMNLLHLCIKETLRMYPPLILLMRKVLKPKIY----------KEYVIPENDIVMVSPAASGRLESVFKNA-NVWDPDRFGPHREED--------- :430
G.sulphuraria   :LNYDV-LSKMNLLHLCIKETLRMYPPLILLMRKVLKPKFY----------KEYVIPENDIVMVSPAASGRLENVFKNP-NAWDPDRFGPNREED--------- :430
G.theta         :IDHEA-IKNMSLLHATVSEALRMQPPLIFVMREVLQDRQF----------KDYTIPKGDVIFLSPSLSGRRPDVWTNP-DSFDPFRFLEPREEH--------- :395
T.trahens       :LNYDV-LEKMDRLHVSIKEGIRLCPPLIFVMRKVKKAFSV----------KGYTVPVGDLAFVSPPVSHRVESIFTNA-ETYDPTRFLPPREED--------- :414
C.sorokiniana   :LDMEV-LNSMDTLHLNIQEALRMNPPLLLVMRYAKEAFKVTT-----SQGKTYTVPKGHIVAASPTFSHRLPHIFNNA-DAYEPDRFLPPREED--------- :425
P.miyajii       :LDFDV-LNELDQLHVCIQEALRLQPPLVLLMRYAKEPFTVTT-----SDGKSYVVPRGDMVATSPSFSHRLQHVFSRA-DAFEPERFLPPREED--------- :422
O.lucimarinus   :LDFDI-LAKMDELHFAVKEALRMHPPLIMLLRMAQVPFEVET-----STGKKYTVPKGHIVATSPAFSHRLDNVYSDP-NEYKPERFREPNPED--------- :399
V.reticuliferus:LSFEA-LSDMEVLHRNITEALRMHPPLLLVMRYAKKPFSVTT-----SNGKTYVIPKGDVVAASPNFSHMLPQIFKNP-EKYDPDRFAPPREEQ--------- :425
G.pectorale     :LSFEA-LSDMEVLHRNITEALRMHPPLLLVMRYAKKPFEVTT-----SDGKSYVIPKGDVVAASPNFAHMLPHIFKNP-KQYDPDRFAPPREEQ--------- :425
G.rivale        :IDHDI-LSEMDTLYRCIKEALRLHPPLIMLLRQSHADFSVQT-----RDGKEYDIPKGHIVATSPAFANRLPHIFKNP-DSYDPDRFAVGREED--------- :415
P.californicum :IDHDI-LSEMDALHRCIKEALRLHPPLIMLLRSSHTDFDVTT-----KEGKEYHIPKGHIVATSPAFANRLPHIYKNP-DSYDPDRFGPDREED--------- :419
V.vinifera      :VDHDI-LSEMDVLYRCIKEALRLHPPLIMLLRSSHSDFSVTT-----KDGKEYDIPKGHIVATSPAFANRLPHIYKDP-ERYDPDRFAVGREED--------- :411
L.tenue         :VDHDV-LSEMDVLYRCIKEALRLHPPLIMLMRQSHSDFSVTT-----QEGKEYDIPKGHIVATSPAFANRLPHIFTEP-DKYDPDRFAPGREED--------- :421
N.fowleri       :LDYDK-VMKMDYLEACMKEALRMYPPLIMIMRMARKPREC----------EQYIIPKGNILVVSPSVAGRCTDTYTNP-DVFDPERLTERKEHE--------- :414
Sea urchin      :VSYDQ-LKDMQLLDHCVKEALRLRPPIMTMMRVAKSPLTY----------KDMTIPAGHQVCVSPTVNQRLKDNWMPGPKEFNPDRFLDESKSN--------- :424
Salmon          :LNFDQ-LKDLTMLDRCLKETLRLRPPIMTMMRMARSPQTV----------AGYTIPAGHQVCVSPTVNHRLQDTWTER-MEFRPDRYLNDNPAA--------- :432
Toad            :LKYDQ-LKDLQVLDRCIKETLRLRPPIMTMMRMARTPQMV----------AGYNIPPGHQVCVSPTVNHRLKDTWVQN-TDFKPDRYLHENPAA--------- :429
Barn owl        :LTYDQ-LRDLSLLDRCLKETLRLRPPIMTIMRMAKTPQTV----------AGYNIPPGHQVCVSPTVNHRLRDSWNDA-LDFKPDRYLQDNPAA--------- :426
Human           :LTYDQ-LKDLNLLDRCIKETLRLRPPIMIMMRMARTPQTV----------AGYTIPPGHQVCVSPTVNQRLKDSWVER-LDFNPDRYLQDNPAS--------- :433
T.brucei        :LNYNNVMDEMPFAERCARESIRRDPPLLMLMRKVMADVKV----------GSYVVPKGDIIACSPLLSHHDEEAFPEP-RRWDPERDEK------------- :407
T.cruzi str.Y B:LNYDNVMDEMPFAERCVRESIRRDPPLLMVMRMVKAEVKV----------GSYVVPKGDIIACSPLLSHHDEEAFPNP-RLWDPERDEK------------- :407
T.cruzi str.Y A:LNYDNVMDEMPFAERCVRESIRRDPSLLMVMRMVKAEVKV----------GSYVVPKGDIIACSPLLSHHDEEAFPNP-RLWDPERDEK------------- :407
C.neoformans    :YRYED-LKELPIMDSIIRETLRMHAPIHSIYRKVLSDIPVPPSLSAPSENGQYIIPKGHYIMAAPGVSQMDPRIWQDA-KVWNPARWHDEKGFAAAA--MVQY :459
S.cerevisiae   :LTYDL-LQEMPLLNQTIKETLRMHHPLHSLFRKVMKDMHVPN--------TSYVIPAGYHVLVSPGYTHLRDEYFPNA-HQFNIHRWNKDSASS--------Y :439
A.fumigatus B   :LTYDN-LQKLDLHAKVIKETLRLHAPIHSIIRAVKNPMAVDG--------TSYVIPTSHNVLSSPGVTARSEEHFPNP-LEWNPHRWDENIAA--------SA :432
N.fischeri B    :LTFDN-LQKLDLHAKVIKETLRLHAPIHSIIRAVKNPMAVDG--------TSYVIPTSHNVLSSPGVTARSEEHFPNP-LEWDPHRWDEDIAA--------SA :432
F.graminearus B:LTYED-LAKLPLNQAIVKETLRLHAPIHSIMRAVKSPMPVPG--------TKYVIPTSHTLLAAPGVSATDSAFFPNP-DEWDPHRWEADSPNFPRMASK--G :439
O.sinensis B    :LTFES-LAKLPLNQAIVKETLRLHAPIHSIMRAVKQPMAVPG--------TKYVIPTSHTLLAAPGVSACDPTFFPNP-DEWDPHRWEADSPNAPTIVRNKVE :443
A.fumigatus A   :LQYKD-LDKLPFHQHVIRETLRIHSSIHSIMRKVKSPLPVPG--------TPYMIPPGRVLLASPGVTALSDEHFPNA-GCWDPHRWENQATK--------EQ :423
N.fischeri A    :LQYKD-LDKLPFHQHVVRETLRLHSSIHSLMRKVKSPLPVPG--------TPYMIPPGRVLLASPGVTALSDEHFPNA-GCWDPHRWENQAAK--------EQ :423
F.graminearus A:LQYSD-LDKLPLLQNVVKETLRVHSSIHSILRKVKRPMQVPN--------SPYTITTDKVIMASPTVTAMSEEYFENA-KTWNPHRWDNRAKE--------EV :418
O.sinensis A    :LQYSD-IDKLPLLQNVIKETLRVHSSIHSVMRKVMRPMQVPN--------SDFTITPDKVLVASPIVTHLSDEYFPNA-QAWDPHRWDDAVET--------D- :427
D.loculata A    :LQYSD-LSKLPLLQNVVKETLRVHSSIHSLMRKVKNPLPVPN--------TEYVITPDKVLLASPIMTHLNEEYFTNP-LAWDPHRWDNIVEV--------E- :423
M.brunneum A    :LQYSD-IDKLPLLQNVIKETLRVHSSIHSIMRKVMRPIPVPK--------TDYVIGPDKVLVSSPIMSHMSEEHFANA-HVWDPHRWENM-ST--------E- :430
                       6  6        E 6R   p6  6 R        v                    6p      sP        5     5 p R
```

```
                      520         *        540         *        560         *        580         *        600         *
A.castellanii   :-------------QAQ-KYRFVGFGAGRHGCMGENFAYLQIKTIWSVLLRNFDIELVG---ELPKPDYTAMVVGPA-HPCLLRYTRK~~~~~~~~~~~~ :486
A.polyphaga     :-------------QAQ-KYRFVGFGAGRHGCMGENFACLQIKTIWSVLLRNFDIELVG---ELPKPDYTAMVVGPA-HPCLLRYTRK~~~~~~~~~~~~ :486
G.partita       :-------------KKA-PFSFIGFGGGRHGCMGEQFAYLQIKTIWTVLIRSFDLEPVG---GLPQPDYNAMVVGPR-PPCLLSYRKKKDSFLDRVPLYS :511
G.sulphuraria   :-------------KKA-PFSFIGFGGGRHGCMGEQFAYLQIKTIWTVLVRSFDLEPIG---DLSQPDYNAMVVGPR-PPCLLKYRKKKDSFLDRVSLYA :511
G.theta         :-------------RKF-SHGWLGFGGGRHRCLGEHFAYVQIKTIWAYLLRNFEMEAVG---PLPSPNYEALVVGPK-HPCLIRYVRRKLPID~~~~~~~ :469
T.trahens       :-------------KKA-RFAFAGFGGGRHGCLGENFAYLQIKTIFSVLLRSYKLKLVD---PFPEQDYTGLVVGPK--PCQVAYERI~~~~~~~~~~~ :482
C.sorokiniana   :-------------KPI-PFSYIGFGGGRHGCMGQNFAYLQIKTIWSVLLRNFDFELLD---PVPEPNYTSMVIMPK--PCRVRFTRKKLQV~~~~~~~~ :497
P.miyajii       :-------------KAV-PFSYIGFGGGRHGCMGSQFAYLQIKTIWSVLLRDFTFELLD---PFPEPDWDSMVIGPK--PCRVRYTRCKLAL~~~~~~~~ :494
O.lucimarinus   :-------------KAQ-FASFIGFGGGRHGCMGETFAYMQIKTIWSILLRNFEFEMVG---KVPEPDYTGMVVGPTAGQCKIRYKRRVL~~~~~~~~~~ :471
V.reticuliferus:--------------NR-PYSFIGFGAGRHACIGQNFAYLQIKSIWSVLLRNFEFELLD---PVPDADYESMVIGPK--SCRVKFTRRKLL~~~~~~~~~ :495
G.pectorale     :--------------NR-NYAFIGFGAGRHACIGQNFAYLQIKSIWSVLLRNFEFELLD---PVPGADYESMVIGPK--PCRVRYTRRKLL~~~~~~~~~ :495
G.rivale        :-------------KASGAFSYISFGGGRHGCLGEPFAYLQIKAIWSHLLRNFELELVS---PFPETDWNAMVVGVK-GNVMVRYKRRELSA~~~~~~~~ :489
P.californicum :-------------KAAGAFSYISFGGGRHGCLGEPFAYLQIKAIWSHLLRNFEFELVS---PFPEIDWNAMVVGVK-GNVMVRYKRRKLVPGDDQ~~~~ :497
V.vinifera      :-------------KVAGAFSYISFGGGRHGCLGEPFAYLQIKAIWSHLLRNFEFELIS---PFPEIDWNAMVVGVK-GKVMVRYKRRVLPVD~~~~~~~ :486
L.tenue         :-------------KAAGAFSYISFGGGRHGCLGEPFAYLQIKAIWSHLLRNFELELVS---PFPEIDWNAMVVGVK-GSVMVRYKRRQLVVD~~~~~~~ :496
N.fowleri       :-------------K-F-KYGAVPFGAGRHKCIGENFALLQVKSIISILLRYFDMEYIG---KIPDPSYTSLVVGPS-PPTRMRYKLRKQQ~~~~~~~~~ :485
Sea urchin      :---------------SEKFSYVPFGAGRHRCIGENFAYVQIKTIWSVMLRIFEFELVDG--YFPGINYQTMIHTPL--NPIIRYKRRTESL~~~~~~~~ :496
Salmon          :---------------GEKFAYVPFGAGRHRCIGENFAYVQIKTIWSTMLRLYEFNLVDG--YFPTINYTTMIHTPH--NPVIRYKRRQH~~~~~~~~~~ :502
Toad            :---------------GEKFAYVPFGAGRHRCIGENFAYVQIKTIWSTMLRLYEFDLVDG--YFPTINYTTMIHTPN--NPIIRYKRRTV~~~~~~~~~~ :499
Barn owl        :---------------GEKFAYVPFGAGRHRCIGENFAYVQIKTIWSTLLRLYEFDLIND--YFPTINYTTMIHTPN--NPVISYKRRSL~~~~~~~~~~ :496
Human           :---------------GEKFAYVPFGAGRHRCIGENFAYVQIKTIWSTMLRLYEFDLIDG--YFPTVNYTTMIHTPE--NPVIRYKRRSK~~~~~~~~~~ :503
T.brucei        :----------------VEGAFIGFGAGVHKCIGQKFGLLQVKTILATAFRSYDFQLLRD--EVPDPDYHTMVVGPTASQCRVKYIRRKAAA~~~~~~~~ :480
T.cruzi str.Y B:----------------VDGAFIGFGAGVHKCIGQKFALLQVKTILATAFREYDFQLLRD--EVPDPDYHTMVVGPTLNQCLVKYTRKKKLPS~~~~~~~ :481
T.cruzi str.Y A:----------------VDGAFIGFGAGVHKCIGQKFALLQVKTILATAFREYDFQLLRD--EVPDPDYHTMVVGPTLNQCLVKYTRKKKLPS~~~~~~~ :481
C.neoformans    :TKAEQVDYGFGSVSKGTESPYQPFGAGRHRCVGEQFAYTQLSTIFTYVVRNFTLKLA--VPKFPETNYRTMIVQPN-NPLVT-FTLRNAEVKQEV~~~~ :550
S.cerevisiae   :SVGEEVDYGFGAISKGVSSPYLPFGGGRHRCIGEHFAYCQLGVLMSIFIRTLKWHY-PEGKTVPPPDFTSMVTLPT-GPAKIIWEKRNPEQKI~~~~~~ :530
A.fumigatus B   :EDDEKVDYGYGLVSKGTNSPYLPFGAGRHRCIGEQFAYLQLGTITAVLVRLFRFRNLPGVDGIPDTDYSSLFSKPL-GRSFVEFEKRESATKA~~~~~~ :524
N.fischeri B    :EDDEKVDYGYGLVNKGTNSPYLPFGAGRHRCIGEQFAYLQLGTITAVLVRLFKFRNLPGVDGVPDTDYSSLFSKPL-GRSFVEFEKRESATKA~~~~~~ :524
F.graminearus B:EDEEKIDYGYGLVSKGSASPYLPFGAGRHRCIGEHFANAQLQTIVAEVVREFKFRNVDGGHTLIDTDYASLFSRPL-EPANIHWERRQ~~~~~~~~~~~ :526
O.sinensis B    :AEDEKIDYGYGMVSKGAASPYLPFGAGRHRCIGEQFANVQLQTITAEVVRIVKLSNVDGGHGLIGTDYASLFSRPL-EPAKVRWERRN~~~~~~~~~~~ :530
A.fumigatus A   :ENDEVVDYGYGAVSKGTSSPYLPFGAGRHRCIGEKFAYVNLGVILATIVRHLRLFNVDGKKGVPETDYSSLFSGPM-KPSIIGWEKRSKNTSK~~~~~~ :515
N.fischeri A    :ENDEVVDYGYGAVSKGTSSPYLPFGAGRHRCIGEKFAYVNIGVILATIVRHLRLFNVDGKKGVPETDYSSLFSGPM-KPSIIGWEKRSKDTSK~~~~~~ :515
F.graminearus A:DTEDVIDYGYGAVSKGTKSPYLPFGAGRHRCIGEKFAYVNLGVIVATLVRNFRLSTIDGRPGVPETDYTSLFSRPA-QPAFIRWERRKKI~~~~~~~~~ :507
O.sinensis A    :TDADMVDYGYGVVSKGTRSPYLPFGAGRHRCIGEKFANVNLCTIICTLVRHLKLDTLDGKSWVPPTDYTSLFSRPA-QPAEVRWARRSPESA~~~~~~~ :518
D.loculata A    :NKEDMVDYGYGAMSKGTKSPYLPFGAGRHRCIGEKFAFLNLVTIVSTLVRNFKFTTIDGKSWVPPTDYTSMFSRPV-QPASIRWERRFPESS~~~~~~~ :514
M.brunneum A    :EKGDMVDYGYGQVSKGTKSPYLPFGAGRHRCIGEKFAYMNLCTIIATLVRNMKFTTVDGSSAVPPTDYTSLFSRPM-QPSTIRWERRANVPSQA~~~~~ :523
                                       FG GrH C6G  Fa  q6  6     R            p   5  6   p      5
```

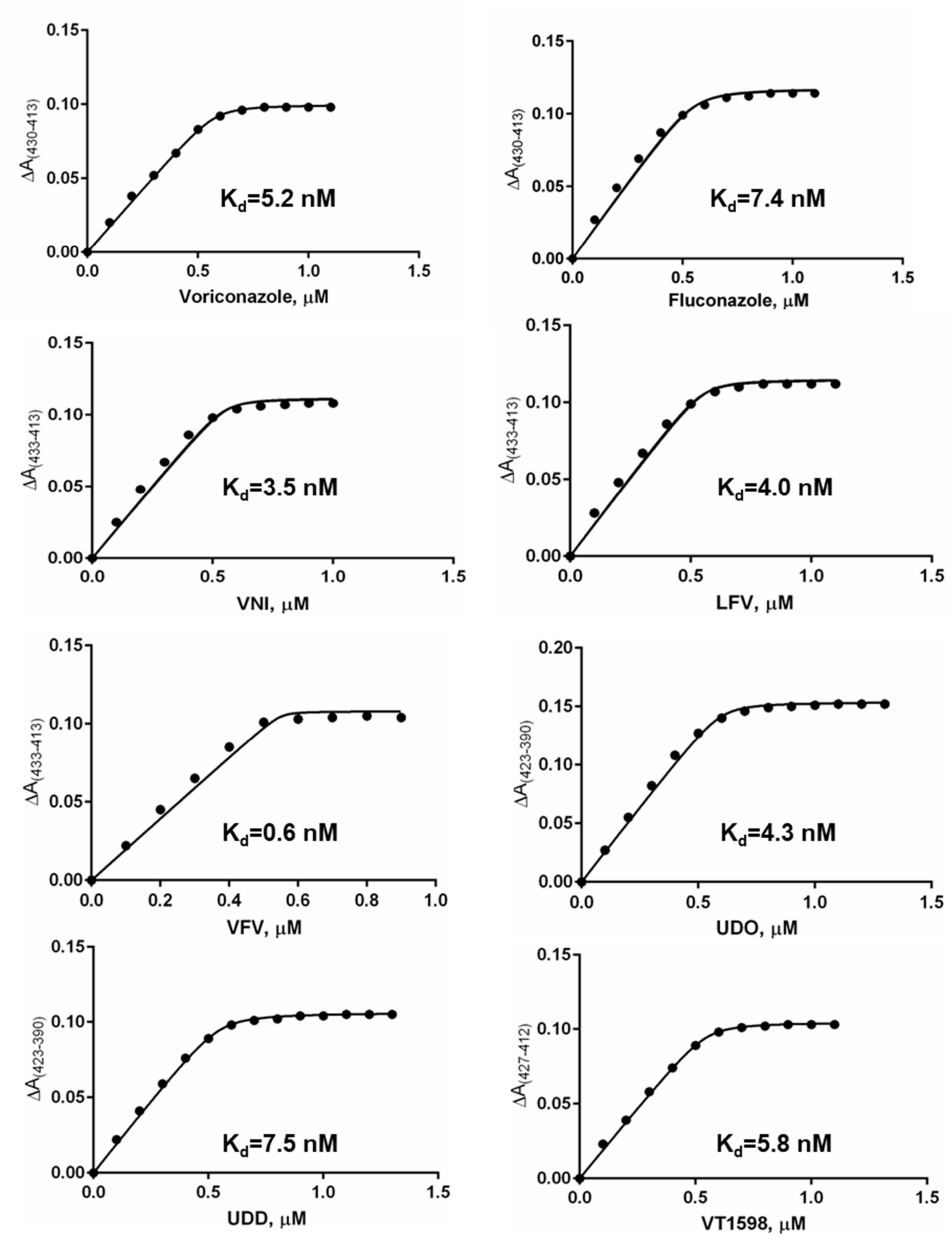

Figure S2. Spectral titration curves for the binding of various heterocyclic ligands to *A. castellanii* CYP51 fitted to the quadratic equation. Two cuvettes each containing 10 mL ~0.5 μM P450 were placed in a spectrophotometer, and the indicated concentrations of the individual compounds were added to the sample cuvette. The values of the spectrally determined apparent dissociation constants are shown. Since the concentration of the protein is much higher than the determined Kds, the values could be not precise.

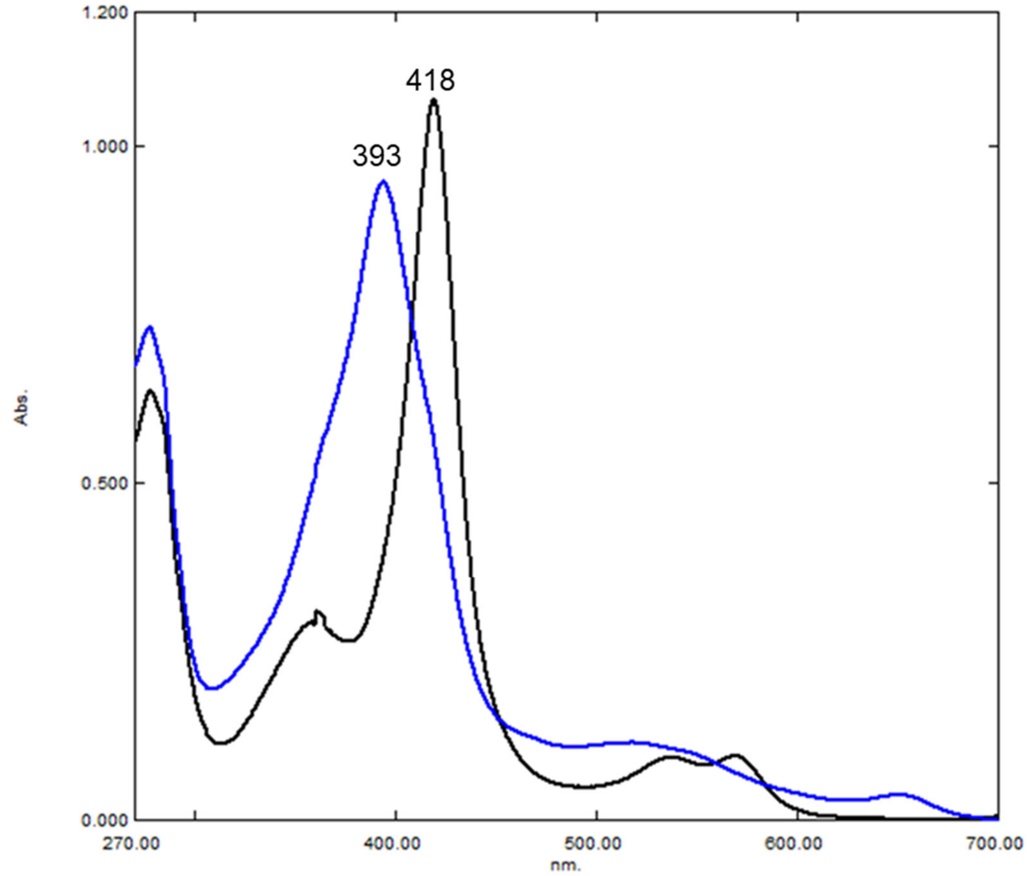

Figure S3. Obtusifoliol-induced spectral response in truncated *A. castellanii* CYP51. Black, low-spin water-bound ferric P450. Blue, >95% high-spin substrate bound ferric P450. The increase in the absorbance at 280 nm is due to the presence of HPCD, which was used to dissolve the sterol.

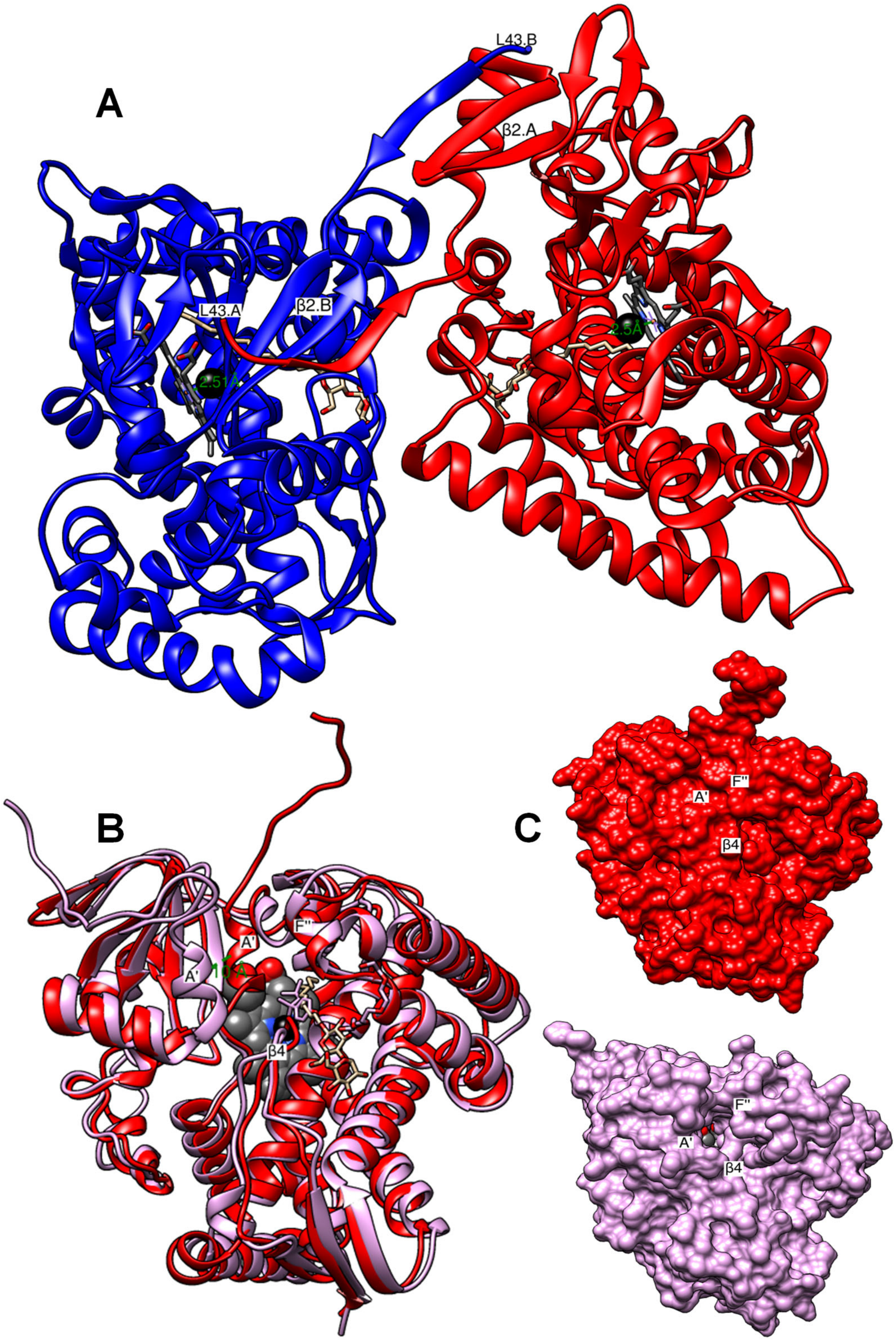

Figure S4. Overview of the crystal structure of *A. castellanii* CYP51 with bound detergent (7UWP). A. The asymmetric unit. Two protein molecules are shown in ribbon representation, red (A) and blue (B). The first N-terminal residue visible in the density map (L43) and the β2-sheet are marled. The heme and n-dodecyl-β-D-maltoside are in stick representation, with the gray and tan carbon atoms, respectively. The iron-coordinated water molecules are shown as black spheres, the distances are in green. **B**. The shift of the A' helix. Molecule A (red) is superimposed with CYP51 from *Methylococcus capsulatus* (6MCW), shown in plum. The secondary structural elements known to form substrate entrance in the CYP51 structures (helices A', F'', and the β4-hairpin) are labeled. **C**. Surface representation (the view and coloring are the same as in B).

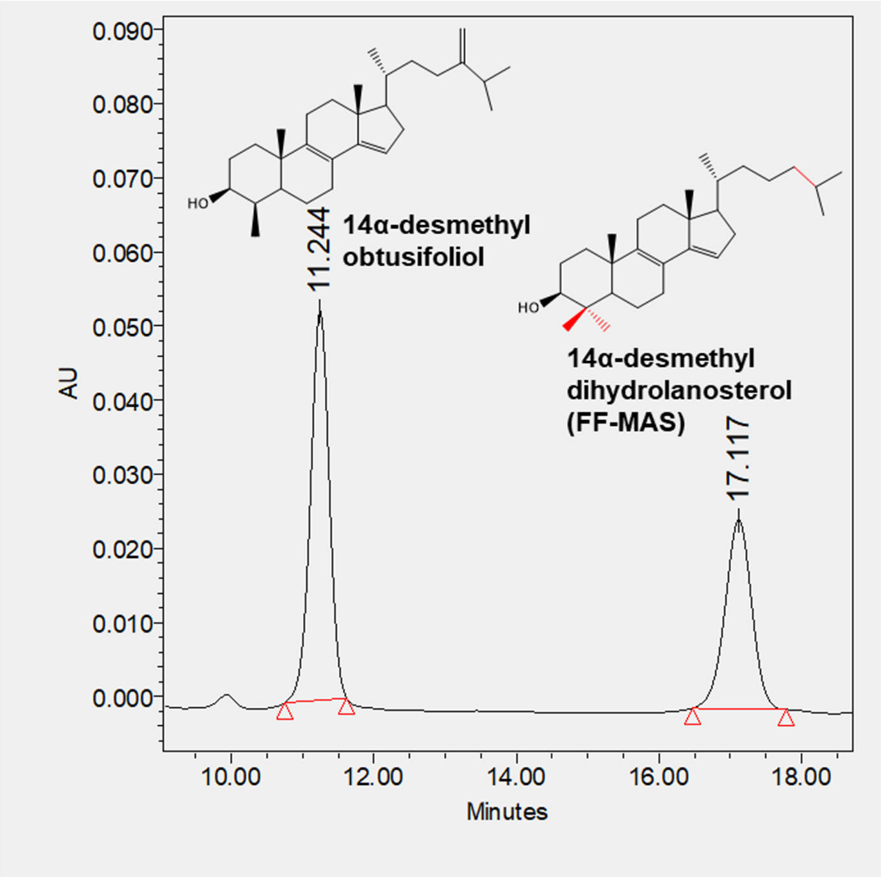

Figure S5. HPLC profile showing the peaks and retention times of the *A. castellanii* CYP51 reaction product (11.2 min) and FF-MAS used as the internal standard (17.1 min). The UV detector set at 250 nm.

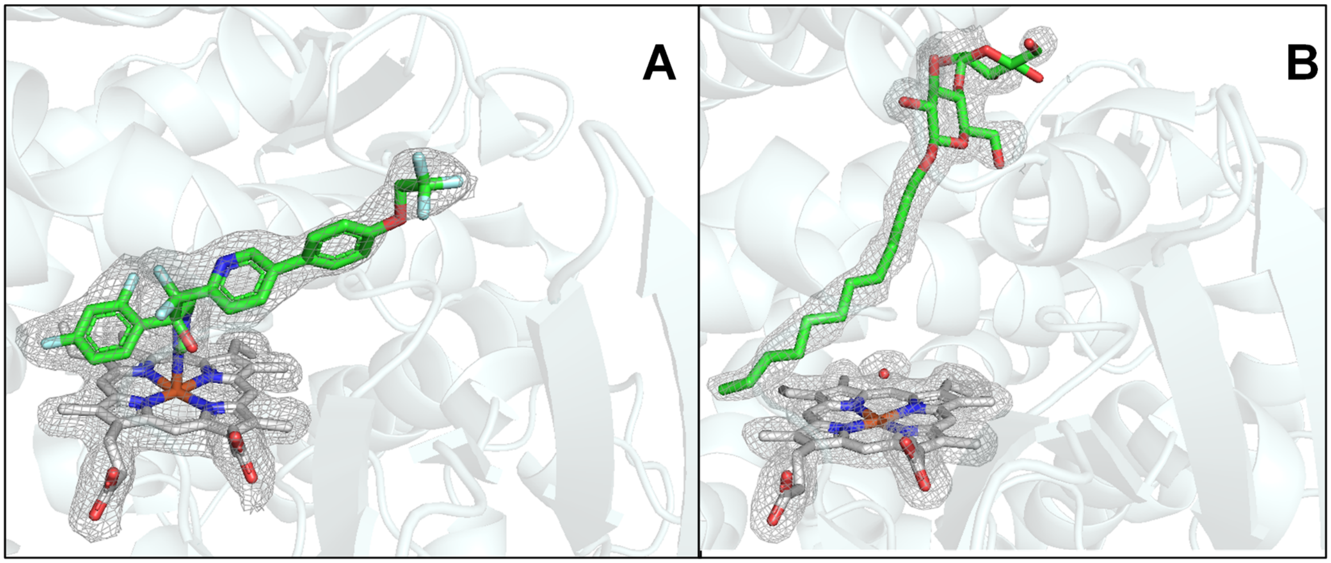

Figure S6. 2Fo-Fc electron density maps for the molecules bound in the CYP51 active site. A. The map for VT1161 and the heme in structure 8EKT, contoured at 1.5 sigma. **B.** The map for the detergent, heme, and the water molecule in structure 7UWP, contoured at 1.1 sigma.

Table S1. Crystallographic Data Collection and Refinement Statistics

PDB ID	7UWP<br>Detergent-bound	8EKT<br>VT1161-bound
Data collection		
Beamline	ID-G	ID-G
Wavelength, Å	0.97856	0.97856
Space group	$P2_12_12_1$	P1
Cell dimensions		
a, b, c, Å	100.4 101.56 122	99.5 99.3 107.9
α, β, γ, °	90.0 90.0 90.0	81.2 84.1 60.1
Molecules per asymmetric unit	2	6
Resolution (upper shell), Å	30.0-1.95 (1.98-1.95)	30.0-2.28 (2.32-2.28)
Solvent content, %	57	59
R_{merge} (upper shell)	0.065 (0.637)	0.040 (1.02)
CC (1/2) (upper shell)	0.990 (0.632)	0.993 (0.539)
I/σ(I) (upper shell)	34 (1.2)	25 (1.7)
Completeness (upper shell), %	89.5 (28.0*)	98.3 (97.6)
Redundancy (upper shell)	9.5 (6.8)	3.9 (3.9)
Refinement		
No. of reflections	91205	118963
R_{work}/R_{free}	0.193/0.225	0.213/0.224-
R.m.s deviations		
Bond lengths, Å	0.005	0.004
Bond angles, °	1.2	0.87
Ramachandran plot		
Favorable/allowed, %	96.7/99.8	96.5/99.8
Outliers, %	0.2	0.2
Average B factor, $Å^2$	25.3	46.0
Model		
No. of atoms	8066	20825
No. of residues per molecule		
Protein (B factor, $Å^2$, A/B/C/D/E/F)	444 (22.5/30.1)	413 (36/39/48/50/51/55)
Heme (B factor, $Å^2$)	1 (9.7/11.3)	1 (12/17/22/21/21/23)
Water (B factor, $Å^2$)	784 (33.9)	90 (36/39/48/50/51/55/39)
Detergent [LMT] (B factor, $Å^2$)	1 (29.3/29.9)	-
Inhibitor [VT1] (B factor, $Å^2$)	-	1 (37/40/43/45/37/52)

*Although the completeness for the upper shell is rather low, the refinement of the structure was performed using the resolution range 30 to 1.95 Å, because all the other parameters were acceptable.

Table S2. VT1161-contacting residues in the CYP51 structures across phyla

Secondary structural element (PDB code)	*A.castellanii* (8EKT)	*T. cruzi* (5AJR)	*C. albicans* (5TZ1)
A’		F48	Y64
B’/B’C	Y114,<br>**F116***,<br><br>F121,<br><br>Y127	Y103<br>M106<br><br>F110<br><br>Y116	Y118,<br>L121<br>T122<br>F126<br>I131<br>Y132
C	**I141**		
FF’’ F’’	V221	F214	F233
I	A290,<br>**L291,**<br>F293,<br>A294,<br>T298	A287<br><br>F290<br>A291<br>T295	G303<br>I304<br>G307<br>G308<br>T311
Kb1-4	L363,<br>L364,<br>**F365,**<br>M367	L356,<br>L357,<br>M358,<br>M360	L376<br>His377<br>S378<br>F380
B4	 M471	 M460	Y505<br>M508
Total	16, no A’ helix	15+Y116 shift	19 +H-bond

In bold are the residues specific to *Acanthamoeba* CYP51s
